# Diagnostic and Prognostic Value of the Venous Excess Ultrasound Grading System (VExUS): A Systematic Review and Meta-Analysis

**DOI:** 10.1097/CCM.0000000000007219

**Published:** 2026-06-09

**Authors:** Peter Klompmaker, Krijna Opschoor, Johan ten Dalen, Ralph de Vries, Cosmin Balan, Denise P. Veelo, Alexander P. J. Vlaar, Pieter R. Tuinman

**Affiliations:** 1 Department of Intensive Care Medicine, Amsterdam UMC, Amsterdam, The Netherlands.; 2 Department of Anaesthesiology, Amsterdam UMC, Amsterdam, The Netherlands.; 3 Amsterdam Leiden IC Focused Echography (ALIFE, www.alifeofpocus.com), Amsterdam, The Netherlands.; 4 Amsterdam Cardiovascular Sciences Research Institute, Amsterdam UMC, Amsterdam, The Netherlands.; 5 Medical Library, Vrije Universiteit, Amsterdam, The Netherlands.; 6 Department of Cardiovascular Anesthesia and Intensive Care Medicine, CC Iliescu Institute for Cardiovascular Diseases, Bucharest, Romania.

**Keywords:** acute kidney injury, fluid tolerance, mortality, venous congestion, venous excess ultrasound

## Abstract

**OBJECTIVES:**

The venous excess ultrasound (VExUS) grading system has gained recognition in acute and critical care medicine as a promising approach to evaluate venous congestion and optimize fluid management. However, the diagnostic accuracy and prognostic value of VExUS remain unclear.

**DATA SOURCES:**

PubMed, Embase.com, Web of Science (Core Collection), and Wiley/Cochrane Library from inception to May 19, 2025, in accordance with the Preferred Reporting Items for Systematic Reviews and Meta-Analyses guidelines and in collaboration with a medical information specialist.

**STUDY SELECTION:**

Study selection was systematically performed by a panel of authors. A total of 32 studies were included, consisting of 3142 patients, with six diagnostic, 22 prognostic studies, and four studies that reported on both diagnostic and prognostic outcomes.

**DATA EXTRACTION:**

Relevant data were extracted by two authors, and the corresponding authors were contacted when necessary.

**DATA SYNTHESIS:**

For diagnostic studies, no meta-analysis could be performed. Studies suggest moderate to good diagnostic accuracy for detecting increased central venous and right atrial pressures (sensitivity 78–95%, specificity 80–90%). Prognostic studies in cardiac patients found an association between VExUS greater than or equal to 2 and acute kidney injury (AKI; odds ratio [OR], 4.44; 95% CI, 2.34–8.43; moderate certainty) and mortality (OR, 3.17; 95% CI, 1.30–7.75, low certainty). For critically ill patients, no associations between VExUS greater than or equal to 2 and AKI (OR, 1.45; 95% CI, 0.70–2.99; very low certainty) and mortality (OR, 1.25; 95% CI, 0.77–2.03; low certainty) were found. Per-patient analysis showed an association between VExUS 3 and mortality for all patients and an association with AKI for cardiac patients.

**CONCLUSIONS:**

Results from this systematic review and meta-analysis suggest that VExUS has moderate to good accuracy to diagnose increased central venous pressure and right atrial pressure. In cardiac patients, higher VExUS grades are associated with AKI and mortality, but in critically ill patients, the association between VExUS grades and outcomes is less clear.

**KEY POINTS**

**Question**: What is the diagnostic accuracy and prognostic value of the venous excess ultrasound (VExUS) grading system?

**Findings**: In this systematic review and meta-analysis, we found moderate to good accuracy in diagnosing increased central venous pressure and right atrial pressure. VExUS greater than or equal to 2 is associated with mortality and acute kidney injury in cardiac patients, while the association in critically ill patients remains less clear.

**Meaning**: VExUS has potential as a tool to guide fluid management, but interventional studies are needed.

In acutely ill patients, fluid management, whether through resuscitation or decongestion, is one of the most common therapeutic interventions. Excessive fluid administration in the ICU has been associated with an increased risk of acute kidney injury (AKI), prolonged need for mechanical ventilation, and heightened mortality (1–4). Patients with acute heart failure and suboptimal decongestion were also shown to experience higher 90-day complication rates (5). Fluid management has traditionally been guided by assessing fluid responsiveness; however, the focus has now shifted toward evaluating fluid tolerance alongside fluid responsiveness. Fluid tolerance denotes the extent to which a patient can tolerate administered fluids without developing organ dysfunction (6). However, a reduced fluid tolerance does not indicate that a patient should not be given fluids, but rather that the risks and benefits of fluid administration should be carefully considered, as with any other therapeutic agent (7). Venous congestion is therefore not a measure of fluid status, but an indicator of the body’s inability to accommodate increased venous return, potentially reflecting a reduced tolerance to fluids.

Therefore, quantifying venous congestion has gained increasing interest. Elevated venous pressure may impair tissue perfusion by increasing pressure within encapsulated organs, such as the kidney, and by reducing the arteriovenous gradient across other vital organs (8–10). This mechanism may explain why some patients experience worse outcomes while others do not (6, 8, 11).

Venous congestion is often assessed using central venous pressure (CVP). However, CVP is a static marker with several technical and interpretive limitations, making it of limited value for detecting venous congestion (12, 13). The venous excess ultrasound (VExUS) grading system has been proposed as a noninvasive bedside alternative for diagnosing systemic venous congestion (14). VExUS is an ultrasound scoring system that integrates measurements of inferior vena cava diameter and Doppler flow patterns in the hepatic, portal, and intrarenal veins to quantify systemic venous congestion. In doing so, VExUS reflects the interplay between cardiac function (particularly right ventricular performance), filling pressures, and volume status (15).

VExUS has also been investigated as a prognostic marker, initially in cardiac populations and more recently in broader patient cohorts (14, 16, 17). Because venous congestion is primarily known to affect the kidneys, early studies explored the association between VExUS grades and AKI. Subsequently, additional research has explored its relationship with outcomes such as mortality and rehospitalization. These studies found varying associations. Therefore, the true relationship between VExUS grades and relevant outcomes remains unknown.

Before VExUS can be incorporated into clinical decision-making, its diagnostic accuracy in detecting venous congestion must be validated, and its association with clinically relevant outcomes such as AKI and mortality must be established. Therefore, we conducted a systematic review and meta-analysis to evaluate the diagnostic and prognostic value of VExUS across different patient populations.

## METHODS

This review is reported according to the Preferred Reporting Items for Systematic Reviews and Meta-Analyses (18). It was registered on PROSPERO on the June 20, 2023, with registry identifier (CRD42023438149).

### Search Strategy

To identify relevant publications, systematic searches of the bibliographic databases PubMed, Embase.com, Web of Science (Core Collection), and Wiley/Cochrane Library from inception to July 2023 were conducted. The search was subsequently updated on December 28, 2025, in collaboration with a medical information specialist.

The following terms were used (including synonyms and closely related words) as index terms or free-text words: “VExUS,” “Hepatic veins,” “Renal veins,” “Hemodynamics,” and/or “Imaging.”

References of the identified articles were searched for relevant publications. Duplicate articles were excluded. Duplicate articles were excluded using EndNote X21.0.1 (Clarivate, Philadelphia, PA), following the Amsterdam Efficient Deduplication method and the Bramer method (19, 20). Full search strategies for all databases can be found in **Supplementary Material** (https://links.lww.com/CCM/H992).

### Selection Process

Two reviewers (P.K., J.v.D.) independently screened all potentially relevant titles and abstracts for eligibility for the first search results using Rayyan (Cambridge, MA). The updated search was also screened by two reviewers (P.K., K.O.). If necessary, the full-text article was checked for the eligibility criteria. Differences in judgment were resolved through a consensus procedure with a third reviewer (P.R.T.). Studies were included when they met the following criteria: VExUS was performed at least once, and relevant prognostic or diagnostic outcomes were reported. Exclusion criteria were: non-English or non-Dutch, used a modified version of VExUS, interventional studies and the following study designs: animal studies, case reports, case series, policy statements, guidelines, conference abstracts, and reviews.

### Endpoints

For diagnostic studies, the following data were extracted: VExUS grades as the index test, sensitivity, specificity, area under the receiver operating characteristic curve, and the reference standard used (e.g., right atrial pressure, CVP).

Data on the following prognostic endpoints were collected: odds ratios (ORs) for AKI and mortality, and the incidence of these endpoints for each possible VExUS grade. Venous congestion was defined as VExUS greater than or equal to 2 for use in the meta-analysis (14). Association of each individual VExUS grade was assessed in a per-patient analysis. Analyses were performed separately for each group of patients (e.g., cardiac, critically ill, or others).

### Data Assessment and Extraction

Full text of selected articles was obtained for further review. Two reviewers (P.K., K.O.) independently evaluated the methodological quality of full-text articles using the Quality Assessment of Diagnostic Accuracy Studies-2 (QUADAS-2) for diagnostic studies and the Quality in Prognostic Studies (QUIPS) for prognostic studies (21, 22). Discrepancies were solved through consensus or discussion with a third reviewer (P.R.T.). Data were extracted by two independent reviewers (P.K., K.O.) and compared. Additional data were retrieved by contacting the corresponding authors if necessary.

Formal certainty of the evidence was performed using the Grading of Recommendations, Assessment, Development, and Evaluation (GRADE) approach. Assessment was done based on risk of bias, imprecision, indirectness, inconsistency, publication bias, large magnitude of an effect, dose-response gradient, and effect of plausible residual confounding (23).

### Statistical Analysis

A meta-analysis was performed if data were available from greater than or equal to 3 studies for each group of patients (e.g., cardiac patients, critically ill patients, or others). Pooled effects were compared using ORs with 95% CIs. *I*^2^ statistics were used to assess heterogeneity among studies. As we anticipated considerable between-study heterogeneity, a random-effects model was used to pool effect sizes. The Paule and Mandel method (24) was used to calculate heterogeneity among studies. Funnel plots were created to assess possible publication bias. Analyses were performed in RStudio (Version 4.4.3, Posit Software, PBC, Boston, MA) using the “meta” and “lme4” packages. ORs and 95% CI of each study were summarized in a forest plot.

A subsequent per-patient analysis, using individual patient data obtained from the study authors, was performed using a mixed-effects model to estimate the OR for each individual VExUS grade, using VExUS 0 as the reference standard. A random intercept was assigned for each study, and a fixed effect was calculated for each VExUS grade. A fixed effect for each VExUS grade was chosen for ease of interpretability and under the assumption that the direction of the effect would be similar in each individual population.

## RESULTS

**Figure 1.**
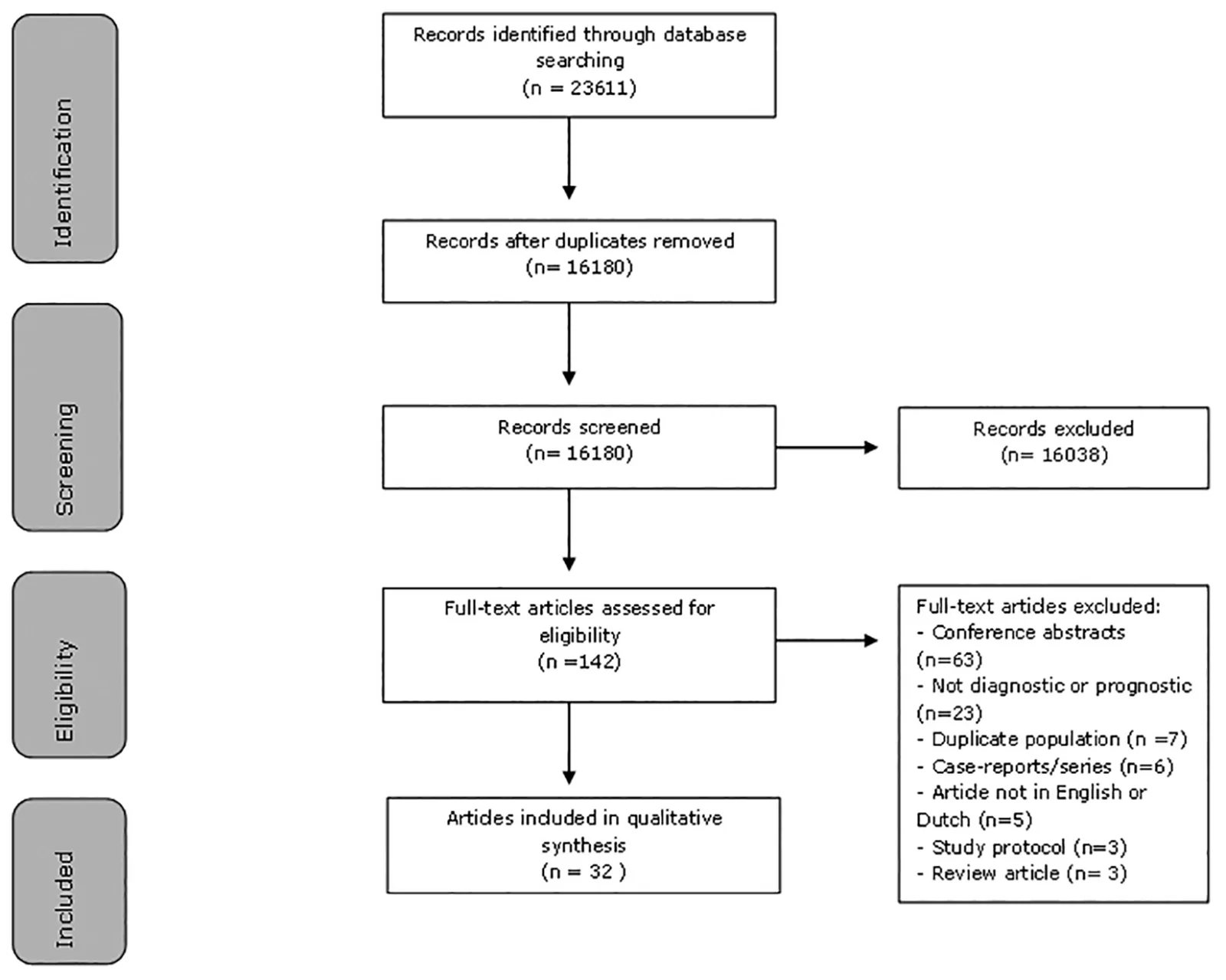
Flowchart of the search and selection procedure of studies.

The flow chart of the literature search and selection process is presented in **Figure 1**. The search generated a total of 23,611 references: 3,994 in PubMed, 8,330 in Embase.com, 10,464 in Web of Science, and 823 in Cochrane Library.

A total of 32 articles were included with a combined total of 3142 patients with a total of six diagnostic, 22 prognostic studies, and four studies that reported on both diagnostic and prognostic outcomes.

The prevalence of the different VExUS grades ranged widely (VExUS 0: 0–69%, VExUS 1: 12–49%, VExUS 2: 4–26%, and VExUS 3: 0–56%) between studies and study populations, but higher grades were more prevalent in patients admitted for cardiac reasons compared with other populations.

### Diagnostic

Of 10 studies with diagnostic outcomes, six involved patients admitted for cardiac reasons, two involved critically ill children, and two involved adult critically ill patients. Individual study characteristics are shown in **Supplementary Table 1** (https://links.lww.com/CCM/H992). Five studies used CVP as a reference standard (*n* = 335), three studies used right atrial pressure (*n* = 203), one study used mean systemic filling pressure (*n* = 60), and one study used the probability of pulmonary hypertension (*n* = 30).

The studies using CVP found poor to moderate agreement and significant associations between higher CVP and higher VExUS grades (25–29). One study reported on the sensitivity (78%; 95% CI, 62–89%) and specificity (82%; 95% CI, 70–90%) of VExUS in detecting high CVP (26). The studies using right atrial pressure as a reference found a high sensitivity (87%) and specificity (90%) for high atrial pressure (> 10 mm Hg) and VExUS greater than or equal to 2, and high sensitivity (95%) and moderate specificity (56%) for low atrial pressure and VExUS 0–1 (30). Furthermore, a significant correlation was found between increasing right atrial pressure and an increase in VExUS grade (Supplementary Tables 1 and **3**, https://links.lww.com/CCM/H992) (31, 32). No meta-analysis was performed because there were either too few studies for each patient group or different reference standards were used; subsequently, no formal GRADE could be assigned.

### Prognostic

Of 26 studies with prognostic endpoints, 14 were in patients admitted for cardiac reasons, 10 were in critically ill patients, one was in noncardiac surgery, and one was in children undergoing cardiac surgery. Individual study characteristics for prognostic studies are shown in **Supplementary Table 2** (https://links.lww.com/CCM/H992).

### Cardiac Patients

Five studies of 14 could not be included in the meta-analysis: one because it did not include the interlobular renal veins in their VExUS grades (33), and four were excluded as the required data were unavailable and additional information could not be obtained (30, 34–36). Of the remaining nine studies, five reported on AKI (*n* = 612) and seven reported on all-cause mortality (*n* = 981) and were included in each meta-analysis, respectively.

For AKI and VExUS greater than or equal to 2, the pooled OR of 4.44 (95% CI, 2.34–8.43; *I*^2^ = 19%) was found. Subsequent per-patient analysis found a significant association between VExUS 2 and 3 with AKI. An overview of the results of all the per-patient analyses is available in **Table 1**. The model converged without warning and met all assumptions. Sensitivity analysis with a leave-one-out approach revealed robust results (**Supplementary Files**, https://links.lww.com/CCM/H992). Certainty of the evidence was moderate (**Table 2**).

**TABLE 1.** Results of the Per-Patient Analysis for Cardiac and Critically Patients

Patient Population	OR (95% CI)
Cardiac	
VExUS and AKI	
0	Reference
1	1.13 (0.72–1.80)
2	2.35 (1.20–4.60)
3	7.54 (4.30–13.22)
VExUS and mortality	
0	Reference
1	1.39 (0.77–2.50)
2	1.39 (0.63–3.03)
3	6.38 (3.37–12.06)
Critically ill	
VExUS and AKI	
0	Reference
1	0.78 (0.53–1.15)
2	0.90 (0.50–1.60)
3	2.17 (0.90–5.52)
VExUS and mortality	
0	Reference
1	1.13 (0.72–1.80)
2	1.22 (0.65–2.31)
3	2.42 (1.13–5.17)

AKI = acute kidney injury, VExUS = venous excess ultrasound.

**TABLE 2.** Grading of Recommendations, Assessment, Development, and Evaluation Assessment

Grade Category	Cardiac AKI	Cardiac Mortality	ICU AKI	ICU Mortality
Risk of bias	—	—	—	—
Inconsistency	—	↓	—	—
Indirectness	—	—	—	—
Imprecision	↓	↓	↓	↓
Publication bias	—	—	—	—
Large magnitude of effect	↑	—	—	—
Dose-response gradient	↑	↑	—	↑
Residual confounding	—	—	—	—
Grading of Recommendations, Assessment, Development, and Evaluation	Moderate	Low	Very low	Low

— = no downgrade or upgrade, ↑ = upgrade of the certainty of evidence, ↓ = downgrade of the certainty of evidence, AKI = acute kidney injury.

**Figure 2.**
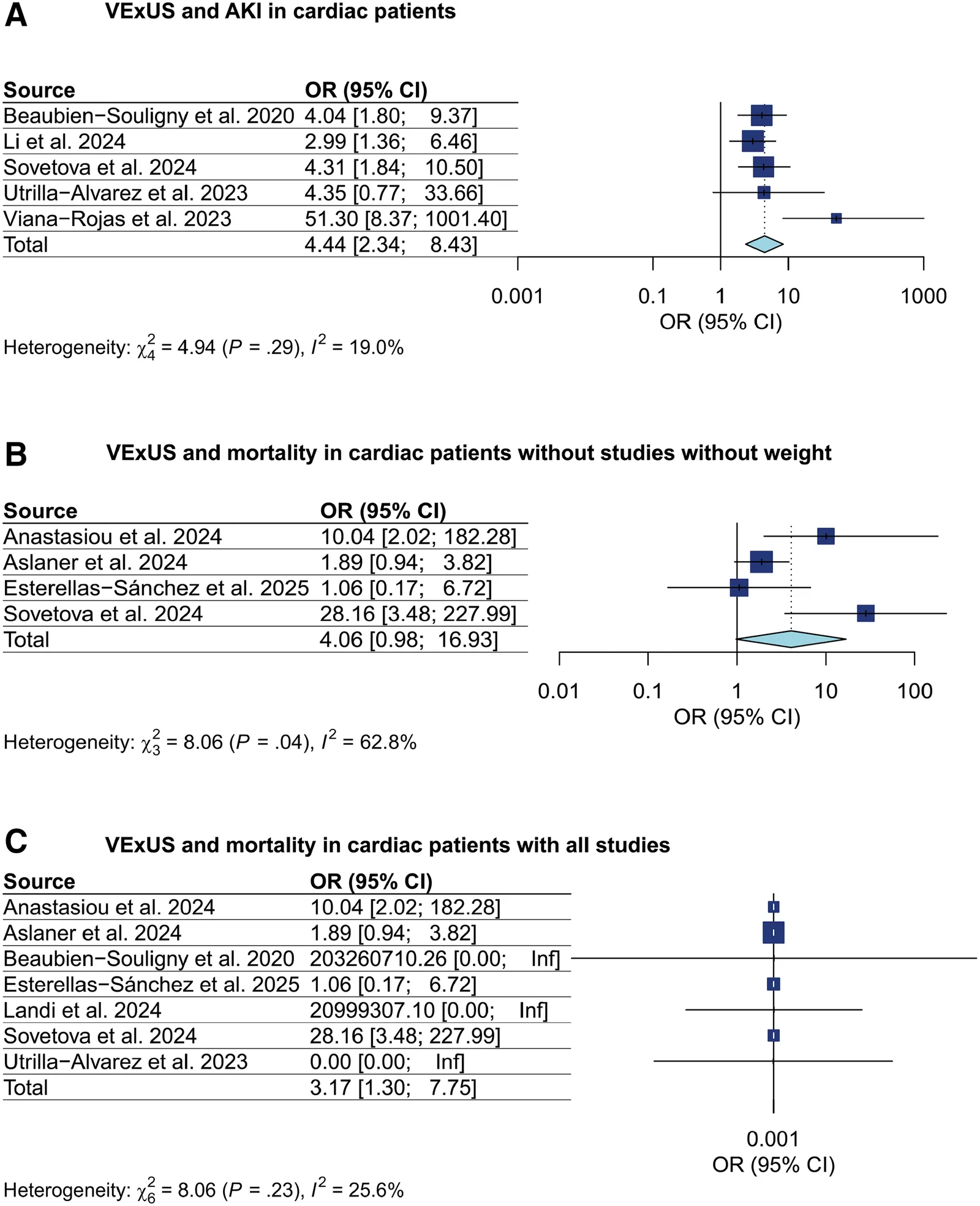
*Forrest plot* for association between acute kidney injury (AKI) and venous excess ultrasound (VExUS) greater than or equal to 2 and mortality and VExUS in cardiac patients. **A**, VExUS and AKI in cardiac patients. **B**, VExUS and mortality in cardiac patients with studies without weight removed. **C**, VExUS and mortality with all studies. OR = odds ratio.

For mortality and VExUS greater than or equal to 2, the pooled OR was 3.17 (95% CI, 1.30–7.75; *I*^2^ = 26%). In three studies, mortality was too low to assign any weight to them in the pooled analysis and caused an unreliable estimate of the OR in these three studies (14, 37, 38). Removing these studies from the meta-analysis showed a pooled OR of 4.06 (95% CI, 0.98–16.93; *I*^2^ = 63%). **Figure 2** shows the forest plot with and without these studies. Per-patient analysis found a significant association between VExUS 3 and mortality (Table 1). The model converged without warning and met all assumptions. Sensitivity analysis with a leave-one-out approach revealed some fragility of the results with a marked effect of a single study. Sensitivity analysis without this study revealed a smaller effect size for VExUS 3 (OR, 4.42; 95% CI, 2.55–8.66; Supplementary Files, https://links.lww.com/CCM/H992). Certainty of the evidence was low (Table 2).

### Critically Ill Patients

Three of 10 studies could not be included in the meta-analysis. Two studies were retrospective cohort studies with a high risk of bias and unclear VExUS protocol. Furthermore, not all the needed data could be obtained, and these studies were therefore excluded from this meta-analysis (39, 40). The required data were unavailable and additional information could not be obtained from one prospective study (41). All necessary data for the per-patient analysis could not be obtained from one study (29). Six studies reported on AKI (*n* = 645), and seven studies (*n* = 758) reported on mortality.

**Figure 3.**
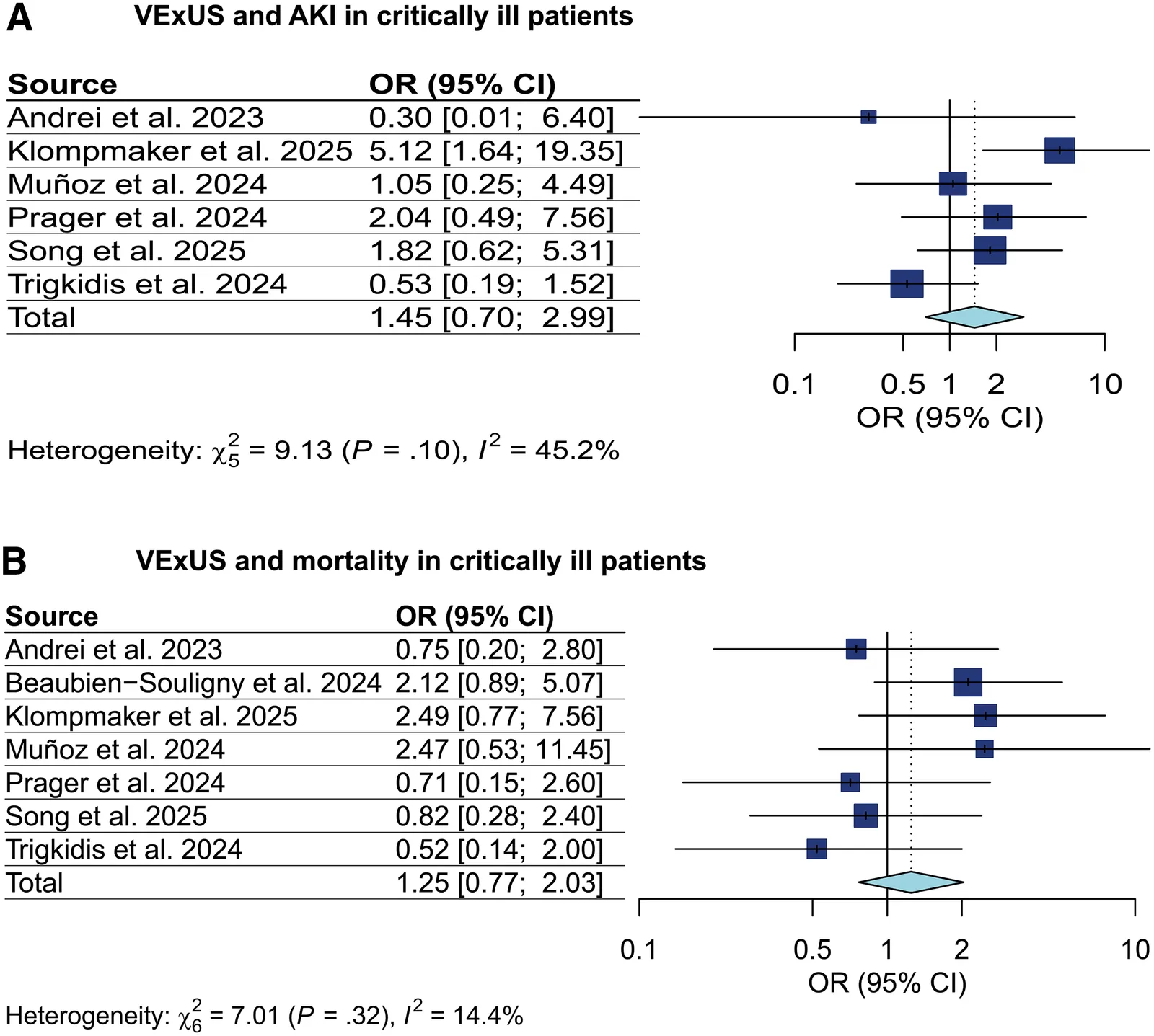
*Forrest plot* for association between acute kidney injury (AKI) and venous excess ultrasound (VExUS) greater than or equal to 2 and mortality and VExUS in critically ill patients. **A**, VExUS and AKI in critically ill patients. **B**, VExUS and mortality in critically ill patients. OR = odds ratio.

For AKI and VExUS greater than or equal to 2 in critically ill patients, the pooled OR was 1.45 (95% CI, 0.70–2.99; *I*^2^ = 45%; **Fig. 3**). Per-patient analysis also showed no significant association between VExUS and AKI (Table 1). The model converged without warning and met all assumptions. Sensitivity analysis with a leave-one-out approach revealed robust results (Supplementary Files, https://links.lww.com/CCM/H992). Certainty of the evidence was very low (Table 2).

The pooled OR for the association between VExUS greater than or equal to 2 and mortality was 1.25 (95% CI, 0.77–2.03; *I*^2^ = 14%; Fig. 3). Per-patient analysis showed a significant association between VExUS 3 and mortality (Table 1). The model converged without warning and met all assumptions. Sensitivity analysis with a leave-one-out approach revealed robust results (Supplementary Files, https://links.lww.com/CCM/H992). Certainty of the evidence was low (Table 2).

### Risk of Bias

**Figure 4.**
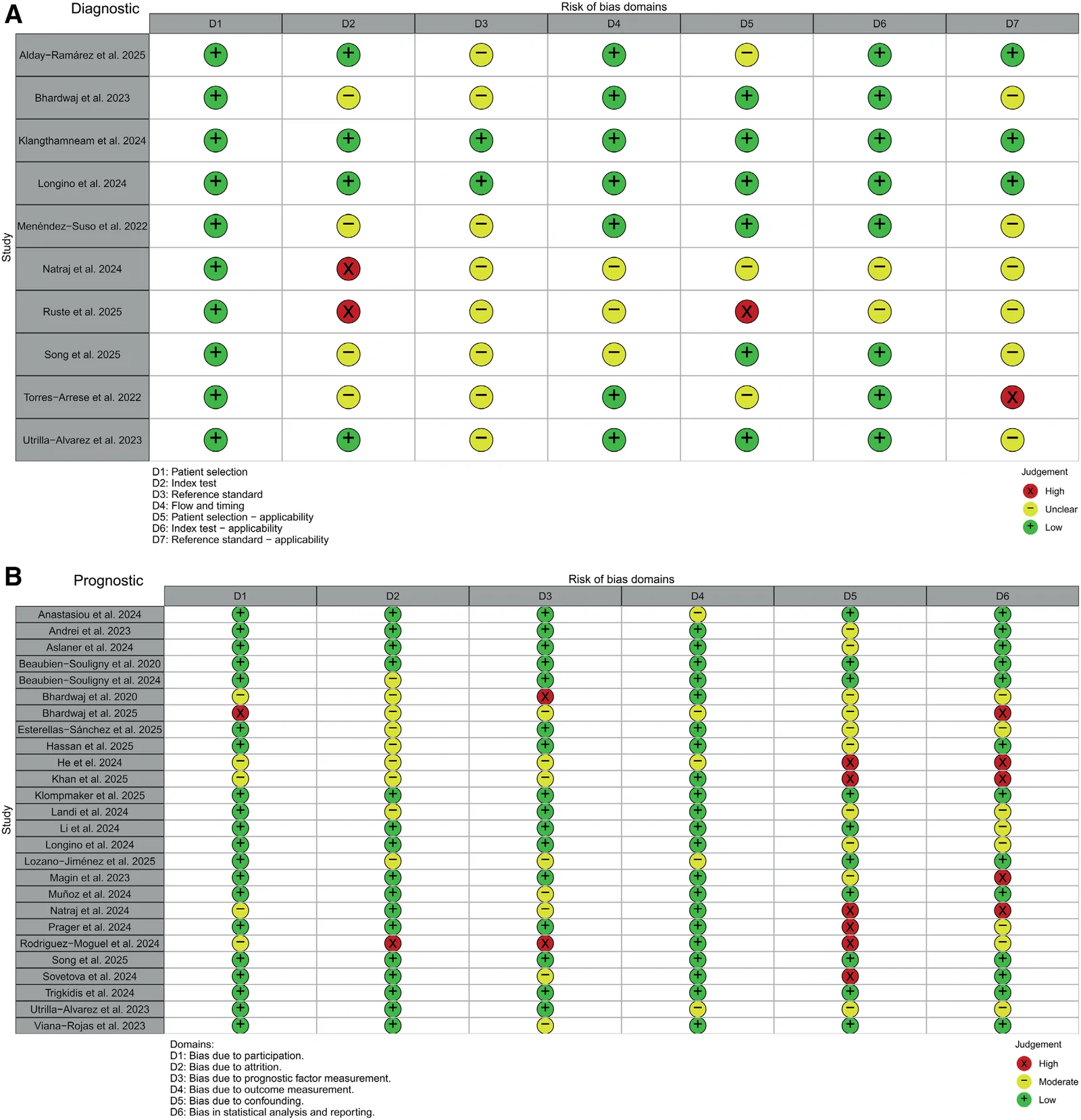
Risk of bias. **A**, Diagnostic studies. **B**, Prognostic studies.

Results of the quality assessment of diagnostic studies are shown in **Figure 4**. Two diagnostic studies showed a high risk of bias due to questions about the performance and timing of the index test and unclear risk of bias in their choice of reference test (27, 28). The other studies showed a low to moderate risk.

Results of the quality assessment of prognostic studies are shown in Figure 4. Four prognostic studies had a high risk of bias, which was mostly due to risks in confounding, reporting and statistical analysis (28, 31, 39, 40). All other studies had a low to moderate risk of bias.

No signs of publication bias were found using funnel plots, as shown in **Supplemental Figure 1** (https://links.lww.com/CCM/H992).

Synthesis of the certainty of the evidence using the GRADE methodology is described in the Supplementary Files (**Supplementary Tables 4–8**, https://links.lww.com/CCM/H992).

## DISCUSSION

The main findings of this systematic review and meta-analysis on the diagnostic accuracy and prognostic value of VExUS show that higher VExUS grades demonstrate moderate to good accuracy in detecting elevated venous and right atrial pressure, although no meta-analysis could be performed to confirm these results. In addition, higher VExUS grades are associated with AKI and mortality in patients admitted for cardiac conditions. Finally, the association between VExUS, AKI, and mortality in critically ill patients remains less clear, with no significant associations identified in the meta-analysis, but a significant association was observed between VExUS grade 3 and mortality in the per-patient analysis.

Our results indicate that the diagnostic role of VExUS in assessing venous congestion remains to be fully established. Several factors contribute to this uncertainty. A meta-analysis of diagnostic accuracy could not be performed. This reflects the absence of a true gold standard for detecting venous congestion and the lack of consensus on which alternative diagnostic tool, such as right atrial pressure, should be used for comparison. Furthermore, VExUS captures more than venous congestion alone, as it reflects the complex interplay between cardiac function, filling pressures, and volume status (15).

It is therefore important to further explore the clinical relevance of the VExUS grading system by examining its relationship with meaningful patient outcomes. Our meta-analysis suggests that higher VExUS grades are consistently associated with adverse outcomes in cardiac populations, whereas associations in other critically ill cohorts are less clear, possibly reflecting smaller effect sizes or limited study power. These differences observed across patient populations may be further explained by the physiologic basis of the VExUS grading system. VExUS reflects the interaction between venous return and cardiac function, rather than intravascular volume alone. Higher VExUS grades likely indicate elevated cardiac filling pressures and impaired right ventricular function, conditions more prevalent in patients with cardiac disease, which may account for the stronger associations observed in cardiac cohorts. In contrast, noncardiac critically ill patients often experience AKI and mortality through multiple, noncardiac pathways, potentially attenuating the relationship between VExUS and clinical outcomes. These findings support a context-dependent interpretation of VExUS grades, emphasizing its role as a marker of cardiac-driven congestion rather than a universal indicator of volume overload (30, 31, 42).

Overall, the observed trend toward increasing risk with higher VExUS grades supports its potential value as an indicator of venous congestion severity and highlights the need for validation in larger, prospective studies.

A previously published systematic review and meta-analysis assessed only the pooled effects of lower VExUS 0–1 vs. higher 2–3 grades (43). Given that our analysis identified the strongest associations specifically for VExUS grade 3, this may explain the less pronounced associations reported in the prior study. The current systematic review and meta-analysis provide a more comprehensive evaluation of the VExUS grading system by additionally including diagnostic accuracy studies, performing analyses at the individual-patient level, and incorporating a larger number of studies and patients.

This study has several limitations. A meta-analysis of the diagnostic accuracy of VExUS could not be performed due to inconsistent reference standards used across studies. In addition, not all necessary data could be obtained from every study, which may have influenced the results. This is especially true for the cardiac mortality analysis since some fragility of the effect size was observed when leave-one-out analysis was applied, although the direction of the effect remained consistent throughout. There was notable clinical and methodological heterogeneity among the included studies. This was limited by separating the analysis by population, and only including studies that used the recommended VExUS grading protocol and by using the earliest available VExUS grades, mostly those at admission or directly after surgery. Furthermore, the studies that investigated interobserver and intraobserver variability found good to excellent agreement (14, 16, 42, 44).

Despite this, substantial statistical heterogeneity was observed in the critically ill patients with AKI, which might have been further explored through subgroup or meta-regression analysis. Unfortunately, the limited number of studies precluded such analyses. This underscores the need for further research in this population. The present study has several strengths. We conducted a comprehensive multidatabase search using broad terminology to capture all iterations of the VExUS grading system, and we obtained additional data by contacting the study authors. Last, we performed separate meta-analyses for distinct patient populations to clarify the prognostic value of VExUS across different clinical settings.

VExUS is a diagnostic tool that has primarily been investigated as a prognostic marker. Although investigating its association with relevant outcomes is important, VExUS should not be used as a prognostic marker for mortality or AKI, as more comprehensive and validated scoring systems already exist (45, 46). Instead, elevated VExUS grades should be interpreted as a marker of the body’s possible inability to accommodate venous return adequately. It indicates that these patients may have a reduced tolerance to increases in preload, for example, by administering fluids. However, this does not mean that fluid administration should be withheld in these patients, but the potential benefits and potentially harmful effects of increasing preload should be weighed carefully. VExUS should therefore be studied in this context, ideally in combination with other measures of fluid tolerance, such as lung ultrasound or diastolic dysfunction indices, to investigate the best way to treat these patients. Trials are needed to establish how best to integrate these markers into fluid management strategies, both during resuscitation and de-resuscitation.

## CONCLUSIONS

In this systematic review and meta-analysis, VExUS demonstrated moderate to good diagnostic accuracy for identifying elevated central venous and right atrial pressures as surrogates for the presence of venous congestion. Higher VExUS grades were associated with AKI and mortality in cardiac populations, whereas these associations were less consistent in other critically ill cohorts. At present, the available evidence is insufficient to support the use of VExUS as a predictive marker for AKI or mortality beyond cardiac populations. Further research, particularly interventional and prospective studies, is needed to clarify the diagnostic performance of VExUS and to establish its optimal role in clinical decision-making.

## Supplementary Material


