# Supplementary material for: Diagnostic and Prognostic Value of the Venous Excess Ultrasound Grading System (VExUS): A Systematic Review and Meta-Analysis
Source: Crit Care Med. 2026 Jun 9;54(9):2419–29. doi: 10.1097/CCM.0000000000007219 (PMC13533470; doi:10.1097/CCM.0000000000007219)
Supplement: Supplementary file 1 [file ccm-54-2419-s001.pdf]

Supplements

Appendix A / Supplementary material

PubMed Search Results – 19 May 2025

Search	Query	Results
#3	#1 AND #2	3,830
#2	"venous congest*"[tiab] OR "vein congest*"[tiab] OR "congested vein*"[tiab] OR "fluid intoleran*"[tiab] OR "fluid overload"[tiab] OR "fluid over load"[tiab] OR "fluid toleran*"[tiab] OR "Venous return"[tiab] OR "Venous Engorgement*"[tiab] OR "engorgement of blood vessel*"[tiab] OR "fluid status"[tiab] OR Hemodynamic*[tiab] OR Haemodynamic*[tiab] OR "volume overload*"[tiab] OR "atrial pressure*"[tiab] OR RAP[tiab] OR "venous pressure*"[tiab] OR CVD[tiab] OR CVP[tiab]	315,719
#1	"venous Doppler*"[tiab] OR VExUS[tiab] OR ((ultrasound*[tiab] OR "ultra sound*"[tiab] OR Doppler*[tiab] OR Ultrasonograph*[tiab] OR Echotomograph*[tiab] OR Sonograph*[tiab] OR Echograph*[tiab] OR Ultrasonic*[tiab] OR Echocardiograph*[tiab] OR "point of care"[tiab]) AND ("Vena hepatica*"[tiab] OR "hepatic vein*"[tiab] OR "portal vein*"[tiab] OR "vena porta"[tiab] OR renal[tiab] OR "vena renalis"[tiab] OR interrenal[tiab] OR intrarenal[tiab]))	41,842

Embase.com Search Results – 19 May 2025

Search	Query	Results
#4	#3 NOT 'clinical trial':dtype	7,859
#3	#1 AND #2	8,061
#2	('venous congest*' OR 'vein congest*' OR 'congested vein*' OR 'fluid intoleran*' OR 'fluid overload' OR 'fluid over load' OR 'fluid toleran*' OR 'fluid intolerance' OR 'Venous return' OR 'Venous Engorgement*' OR 'engorgement of blood vessel*'	478,419

Search	Query	Results
	OR 'fluid status' OR Hemodynamic* OR Haemodynamic* OR 'volume overload*' OR 'atrial pressure*' OR RAP OR 'venous pressure*' OR CVD OR CVP):ti,ab,kw	
#1	'venous Doppler*':ti,ab,kw OR VExUS:ti,ab,kw OR ((ultrasound* OR 'ultra sound*' OR Doppler* OR Ultrasonograph* OR Echotomograph* OR Sonograph* OR Echograph* OR Ultrasonic* OR Echocardiograph* OR 'point of care') AND ('Vena hepatica*' OR 'hepatic vein*' OR 'portal vein*' OR 'vena porta' OR renal OR 'vena renalis' OR interrenal OR intrarenal)):ti,ab,kw	79,582

Web of Science (Core Collection) Search Results – 19 May 2025

Search	Query	Results
#3	#1 AND #2	10,182
#2	TS=('venous congest*' OR 'vein congest*' OR 'congested vein*' OR 'fluid intoleran*' OR 'fluid overload' OR 'fluid over load' OR 'fluid toleran*' OR 'Venous return' OR 'Venous Engorgement*' OR 'engorgement of blood vessel*' OR 'fluid status' OR Hemodynamic* OR Haemodynamic* OR 'volume overload*' OR 'atrial pressure*' OR RAP OR 'venous pressure*' OR CVD OR CVP)	470,153
#1	TS=('venous Doppler*' OR VExUS OR ((ultrasound* OR 'ultra sound*' OR Doppler* OR Echograph* OR Ultrasonograph* OR Echotomograph* OR Sonograph* OR Ultrasonic* OR Echocardiograph* OR 'point of care') AND ('Vena hepatica*' OR 'hepatic vein*' OR 'portal vein*' OR 'vena porta' OR renal OR 'vena renalis' OR interrenal OR intrarenal)))	58,377

Wiley/Cochrane Library Search Results – 19 May 2025

Search	Query	Results
#3	#1 AND #2	784
#2	((venous NEXT congest*) OR (vein NEXT congest*) OR (congested NEXT vein*) OR (fluid NEXT intoleran*) OR (fluid NEXT overload) OR (fluid NEXT over NEXT load) OR “fluid toleran*” OR (Venous NEXT return) OR (Venous NEXT Engorgement*) OR (engorgement NEXT of NEXT blood NEXT vessel*) OR (fluid NEXT status) OR Hemodynamic* OR Haemodynamic* OR (volume NEXT overload*) OR (atrial NEXT pressure*) OR RAP OR (venous NEXT pressure*) OR CVD OR CVP):ti,ab,kw	61,711
#1	(venous NEXT Doppler*):ti,ab,kw OR VExUS:ti,ab,kw OR ((ultrasound* OR ultra-sound* OR Doppler* OR Ultrasonograph* OR Echotomograph* OR Sonograph* OR Echograph* OR Ultrasonic* OR Echocardiograph* OR "point of care") AND ((Vena NEXT hepatica*) OR (hepatic NEXT vein*) OR (portal NEXT vein*) OR (vena NEXT porta) OR renal OR (vena NEXT renalis) OR interrenal OR intrarenal)):ti,ab,kw	4,817

Supplementary table 1. Study and patient characteristics of diagnostic studies

Study	Study design	Setting/patient (n=)	Age Mean/median ± SD or [IQR]	Sex n(%)	CKD	Low LVEF* or CHF† n(%)	Ventilated n(%)	VExUS n(%)	Reference standard
**Alday-Ramírez et al. 2025**	Crossectional single centre observational study	Patients undergoing right heart catheterisation for suspected pre-capillary pulmonary hypertension (49)	49±14	14(29%)	-	Na	-	0=28(57%)<br>1=12(24%)<br>2-3=9(18%)	RAP
**Bhardwaj et al. 2023**	Prospective observational single center,	Cardiac surgery (107)	56 ± 13	78 (73%)	NA	NA	56 (52%)	0=63 (59%)<br>1=15 (14%)<br>2=14 (13%)<br>3=15 (14%)	CVP
**Klangthamneam et al. 2024**	Crosssectional observational single center	RHC outpatient (73)	57 ± 17	32 (44%)	NA	9 (12%)*	0	0=37 (51%)<br>1=9 (12%)<br>2= 9 (12%)<br>3=18 (25%)	RAP
**Longino et al. 2024**	Crosssectional observational single center	RHC in- (AHF) and outpatient (81)	61 [54-70]	55 (69%)	NA	45 (56%)*	0	0= 30 (37%)<br>1= 25 (31%)<br>2= 15 (19%)<br>3= 11(14%)	RAP
**Menéndez-Suso et al. 2022**	Prospective observational single center,	Pediatric ICU (33)	12 months [4.1-100.6]	20 (61%)	NA	NA	19(58%)	0=19(58%)<br>1=10(30%)<br>2=2(6%)<br>3=2(6%)	CVP

**Natraj et al. 2024**	Prospective observational single center	Children 1 month to 17, cardiac surgery (43)	14 months [6-47]	19 (44%)	NA	NA	0	0=0 (0%)<br>1=10 (23%)<br>2=12 (28%)<br>3=19 (44%)	CVP
**Ruste et al. 2025**	Post hoc analysis of prospective observational single center study	ICU with hemodynamic instability & IVC>20mm (32)	70 [62-77]	22 (69%)	NA	NA	19 (60%)	VExUS 0 not measured<br>1=6 (19%)<br>2=13 (41%)<br>3=13 (41%)	CVP or dVR
**Song et al. 2025**	Prospective observational multi centre cohort study	Patients admitted to a tertiary centre ICU with sepsis (n=108)	74(61-84)	77(71%)	12(11%)	HF 14(13%)	86(80%)	0=53(49%)<br>1=36(33%)<br>2=16(15%)<br>3=3(3%)	CVP
**Torres-Arrese et al. 2022**	Prospective observational single center	AHF (30)	79 ± 13.4	15 (50%)	8 (27%)	3 (10%)	0	0=8 (27%)<br>1=11 (37%)<br>2=6 (20%)<br>3=5 (17%)	Probability of pulmonary hypertension (low, intermediate or high) based on: max velocity tricuspid insufficiency , ratio of right ventricle to left ventricle, pulmonary artery and inferior vena cava diameter
**Utrilla-Alvarez et al. 2023**	Cross-sectional observational single center	Cardiac surgery (60)	60 [51-68]	35 (58%)	4 (7%)	21 (35%)†	0	24h<br>0=25 (42%)<br>1=29 (48%)<br>2=4 (7%)<br>3=2 (3%)	mean systemic filling pressure calculated by invasive Anderson method

CKD = Chronic kidney disease, CVP = Central Venous Pressure, RAP = Right Atrial pressure, AHF = Acute Heart Failure, LVEF= Left ventricular ejection fraction, CHF = congestive heart failure

Supplementary table 2. Study and patient characteristics of prognostic studies

study	Study design	Setting/Patients (n=..)	Age mean± SD or median (IQR)	Sex n (%)	CKD n(%)	Cardiac function n(%)	Ventilated n(%)	Outcome	VExUS n(%)	AKI n(%)	Mortality n(%)
**Anastasiou et al. 2024**	Prospective observational single center	Acute heart failure in the ward (290)	72.4 ± 13.2	177 (61%)	38 (13%)	NYHA 3-4: 92 (32%)	0 (0%)	in hospital mortality	0=42 (14%)<br>1=60 (21%)<br>2=74 (26%)<br>3=114 (39%)	NA	18 (6.2%)
**Andrei et al. 2023**	Prospective observational multicentre, four tertiary centers	Mixed ICU (145)	64 ± 15	86 (59%)	15 (10%)	CHF 21 (15%)	NA	AKI (KDIGO) & 28-day mortality	0=62(43%)<br>1=54(37%)<br>2=23(16%)<br>3=6(4%)	68 (47%)	39 (28%)
**Aslaner et al. 2024**	Post-hoc analysis of prospective observational single center study, tertiary care	Emergency department with AKI (246)	74 [65-83]	111 (45%)	69 (28%)	CHF 54 (22%)	0 (0%)	6-month mortality	0=169 (69%)<br>1=39 (16%)<br>2=15(6%)<br>3=23(9%)	NA	97 (39%)
**Beaubien-Souligny et al. 2024**	Prospective cohort multicenter , five tertiary centers	Mixed ICU with AKI (113)	67 [56-73]	82 (73%)	22 (17%)	CHF 15(13%)	78(69%)	MAKE30, 30-day mortality, 90-day mortality	0-1= 83 (73%)<br>2 = 14(12%)<br>3 = 16 (14%)	NA	30-day mortality 35 (31%)<br>90-day mortality 40(35%)
**Beaubien-Souligny et al. 2020**	Post-hoc analysis of prospective observational single center study, tertiary care	Cardiac surgery (145)	66 ± 13	107 (73.8%)	37 (26%)	Low LVEF 31 (21.4%)	0 (0%)	AKI	0= 52 (36 %)<br>1= 61 (42%)<br>2= 15 (10%)<br>3= 17 (12%)	49 (34%)	1 (1%)
**Bhardwaj et al. 2020**	Prospective observational single center	ICU, cardiorenal syndrome with AKI (30)	60 ± 16	21 (70%)	NA	NA	23 (77%)	AKI clearance	0= 52 (36 %)<br>1= 61 (42%)<br>2= 15 (10%)<br>3= 17 (12%)	30 (100%)	NA

**Bhardwaj et al. 2025**	Prospective observational single center cohort study	Patients admitted to a tertiary cardiac ICU with acute heart failure(no mechanical ventilation or RRT (111)	58 ± 14	88(79%)	23(21%)	Median LVEF 35%(25-45) (100%)	10(9%)	90 day mortality, MAKE-90,AKI	Not reported	60(54%)	36(32%)
**Esterellas-Sánchez et al. 2025**	Prospective observational single center cohort study	Acute or decompensated heart failure (90)	85 ± 6	47(52%)	32(36%)	LVEF<40% 30(33%)	-	In hospital morality, 3 month cardiovascular mortality	0= 16(20%) 1=33(41%) 2=19(24%) 3=12(15%)	-	In hospital mortalty 6(7%) 3 month cardiovascular mortality 9(10%)
**Hassan et al. 2025**	Prospective observational single center cohort study	Patients with reduced LVEF discharged from tertiary hospital (109)	57(51-65)	89(82%)	26(24%)	reduced LVEF 109(100%)	-	90 day All-cause mortality after discharge, Worsening renal failure >50% increase in creatinin	Not reported	15(14%)	Not reported
**He et al. 2024**	Retrospective single center cohort study	ICU, acute hyperlipide pancreatitis (110)	AKI 37 ± 5 Non-AKI 39 ± 10	79 (71.8 %)	0	NA	0 (0%)	AKI	Not reported	23 (21%)	NA
**Khan et al. 2025**	Prospective observational single center	ICU, noncardiac patients (30)	Non-AKI 43 ± 18.6 AKI 42 ± 19	14 (47%)	0	NA	NA	AKI within 6 days; 30-day mortality	1=23(77%) 2=6(20%) 3=1(3%)	22 (73%)	14 (47%)

**Klompmaker et al. 2025**	Prospective observational cohort, single tertiary care	mixed ICU (138)	67.5 [56-75]	92 (67%)	9 (6%)	CHF 9 (6%)	82 (59%)	30-day mortality; MAKE30; AKI KDIGO >2	0 = 57 (41%) 1 = 66 (48%) 2 = 10 (7%) 3 = 5 (4%)	53 (38%)	32 (23%)
**Landi et al. 2024**	Prospective observational single center, secondary care	Emergency department with AHF (50)	83 [79-88]	29 (58%)	NA	LVEF <40%; 12 (24%)	0 (0%)	In hospital mortality & Hospital readmission	0=0 (0%) 1=11(22%) 2=11(22%) 3=28(56%)	NA	6 (15%)
**Li et al.2024**	Prospective observational cohort single center	Cardiac surgery (230)	60 [50-68]	135 (59%)	NA	NYHA classification* 1=47(20%) 2=90(39%) 3=74(32%) 4=19(8%)	0 (0%)	AKI	0=154(67%) 1=43(19%) 2=10(4%) 3=23(10%)	53 (23%)	NA
**Longino et al. 2024**	Cross-sectional observational cohort single center	In- and outpatient right heart catheterisation (81)	61 [54-70]	55(69%)	NA	Low LVEF45 (56%)	0 (0%)	AKI	0= 30 (37%) 1= 25 (31%) 2= 15 (19%) 3= 11(14%)	NA	NA
**Lozano-Jiménez et al. 2025**	Prospective observational multi center cohort study	Patients admitted for heart failure in tertiary care centre (120)	75 ± 15	75 (62%)	41(34%)*	LVEF<40% 39(33%)	-	All-cause mortality and hospital readmission	Not reported	-	16(13%)
**Magin et al. 2024**	Prospective observational pilot single center	Non-cardiac surgery (69)	62 [48-67]	46 (67%)	NA	NA	0 (0%)	AKI, 30-day hospital readmission, 30-day mortality	0=37(56%) 1=22(33%) 2=7(11%)	11 (16%)	1 (1%)

**Munoz et al. 2024**	Prospective cross-sectional multicenter , three tertiary centers	Mixed ICU (90)	63 [45-71]	46 (51%)	NA	NA	90 (100%)	AKI & 28-day mortality	0 = 45 (50%)<br>1 = 37 (41%)<br>2 = 6 (7%)<br>3 = 2 2(%)	44 (49%)	19 (21%)
**Natraj et al. 2024**	Prospective observational single center	Children 1 month to 17, after cardiac surgery (43)	14 months [6-47]	19 (44.2%)	NA	NA	0 (0%)	AKI (RIFLE criteria)	0=0 (0%)<br>1=10 (23%)<br>2=12 (28%)<br>3=19 (44%)	18 (42%)	NA
**Prager et al. 2024**	Prospective observational multicenter, two centers	ICU, septic shock (75)	64 [56 – 74]	36(48%)	8 (11%)	CHF 5 (7%)	47 (63%)	MAKE30; 30-day mortality; 30-day RRT	0=33(44%)<br>1=28(38%)<br>2=8(11%)<br>3=6(8%)	RRT 10 (14%)	20 (27%)
**Rodríguez-Moguel et al. 2024**	Retrospective single center	ICU with COVID (69)	51 ± 16	60 (75%)	2 (3%)	NA	69 (100%)	60-day mortality	0=34(49%)<br>1=19(28%)<br>2=12(17%)<br>3=4(6%)	NA	14 (20%)
**Song et al. 2025**	Prospective observational multi center cohort study	Patients admitted to a tertiary center ICU with sepsis (108)	74(61-84)	77(71%)	12(11%)	HF 14(13%)	86(80%)	30 day mortality, AKI according to KIDIGO criteria	0=53(49%)<br>1=36(33%)<br>2=16(15%)<br>3=3(3%)	58(54%)	30(28%)
**Sovetova et al. 2024**	Prospective observational single center	AHF in ICU (100)	75 [64-81]	60 (60%)	NA	Non ischemic cardiomyopathy 8(8%)	0 (0%)	Worsening renal failure & 30-day mortality	0=36(36%)<br>1=26(26%)<br>2=12(12%)<br>3=26(26%)	37 (37%)	13 (13%)
**Trigkidis et al. 2024**	Prospective observational single center, tertiary care	Mixed ICU (89)	62 [51-72]	55 (62%)	3 (3%)	NA	NA	MAKE30, 30-day mortality, AKI	0=22(25%)<br>1=43(48%)<br>2=20(22%)<br>3=4(4%)	60 (67%)	17 (19%)
**Utrilla-Alvarez et al. 2023**	Prospective cross-sectional observational single center	Cardiac surgery (60)	60 [51-68]	35 (58%)	4 (7%)	CHF 21 (35%)	0 (0%)	AKI	0=25 (42%)<br>1=29 (48%)<br>2=4 (7%)<br>3=2 (3%)	21 (35%)	NA

**Viana-Rojas et al. 2023**	Prospective observational single center	Emergency department with ACS (77)	58 ± 10	66 (85.7)	5 (6%)	NA	0 (0%)	AKI (KDIGO)	0=46 (60 %)<br>1=21 (27%)<br>2=4(5%)<br>3=6 (8%)	19 (25%)	NA

NA = not available, ICU= intensive care unit, AKI = acute kidney injury, CHF= congestive heart failure, LVEF= left ventricular ejection fraction, NYHA = New York Heart Association Functional Classification, RRT= renal replacement therapy, ACS= acute coronary syndrome

Supplemental table 3 outcomes of diagnostic studies

Study	Diagnostic accuracy outcomes	Other outcomes
**2022, Menendez**		Associatie CVP en VExUS; hoeveel hoger CVP dan CVP bij VExUS 0<br>Diff in CVP from 0<br>1=3.43 (1.10-5.76) SE 1.14 p0.005<br>2=5.13 (0.7-9.56) SE 2.17 p0.025<br>3=9.63(5.20-14.6) SE 2.17 p<0.001
**2023, Bhardwaj**	Total (107)<br>Sens 78% (62.4-89.4),<br>spec 81.8 (70.39-90.24)<br>PPV 72.7 (60.9-82)<br>NPV 85.7 (76.9-91.5)<br>accuracy 80.37 (71.5-87.4)<br><br>Ventilated (56)<br>Sens 83.3 (58.5-96.4)<br>Spec 92.1 (78.6-98.3)<br>PPV 83.3 (62.2-93.7)<br>NPV 92.1 (80.5-97)<br>Acc 89.2 (78.1-95.9)<br><br>Nonventilated (51)<br>Sens 73.9 (51.5-89.7)<br>Spec 67.8 (47.6-84.1)<br>PPV 65.3 (51.1-77.3)<br>NPV 76 (60.3-86.8),<br>accur 70.5 (56.1-82.5)	Kappa 0.75 vent, kappa 0.41 in non ventilated
**Torres Arrese et al. 2022**		PH in AHF; AUC 80.1; p0.017
**Klangthamneam et al. 2024**		VExUS to right atrial pressure median mmhg (IQR)<br>0=6 (4-8)<br>1=9(7-13)<br>2=11(9-11)<br>3=15.5(11-19) p0.001
**Longino et al. 2024**	VExUS 0-1 - RAP <7mmHG; sens 0.95 (0.64-1); spec 0.56 (0.46-0.86)<br>VExUS >=2 - RAP >10; sens 0.87 (0.75-0.97); spec 0.9 (0.73-0.96)<br><br>AUC RAP > 10mmHg 0.9 (0.83-0.97)<br>AUC RAP <7 mmHg 0.79 (0.7-0.87)	

**Ruste 2025**		Correlaties between:<br>Vexus and CVP:P0.006<br>Pmsa P0.004<br>dVR 0.934
**Utrilla Alvarez et al. 2023**		Baseline<br>0=15-20 rho 0.22; p0.08<br><br>24h<br>1=20-25<br>2=25-30<br>Rho 0.72; p0.00<br><br>48h<br>3=>30<br>Rho 0.86; p0.00
**Alday-Ramírez et al.2025**	RAP > 10 mmHg<br>(AUC 0.93, $p < 0.001$)	correlation<br>between VExUS score and RAP (R = 0.70,<br>$p < 0.001$)
**Natraj et al. 2024**		CVP >8mmHg<br>4/12 V1; 11/12 V2; 19/19 V3<br>P<0.001

Supplemental Figure 1. Funnel plots for publication bias assessment

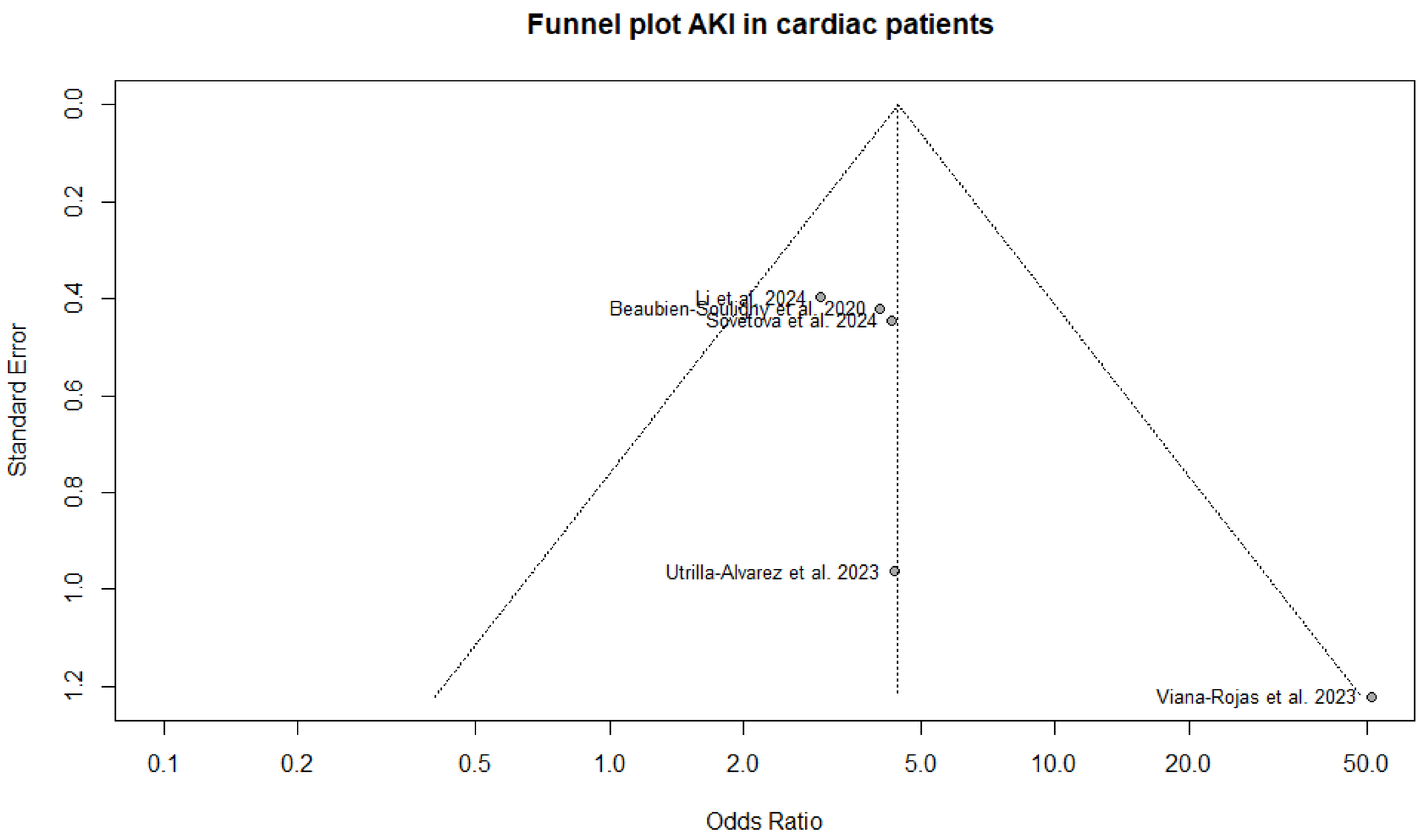

Funnel plot AKI in cardiac patients
Standard Error
0.0
0.2
0.4
0.6
0.8
1.0
1.2
Li et al. 2024
Beaubien-Souligny et al. 2020
Sovetova et al. 2024
Utrilla-Alvarez et al. 2023
Viana-Rojas et al. 2023
0.1
0.2
0.5
1.0
2.0
5.0
10.0
20.0
50.0
Odds Ratio

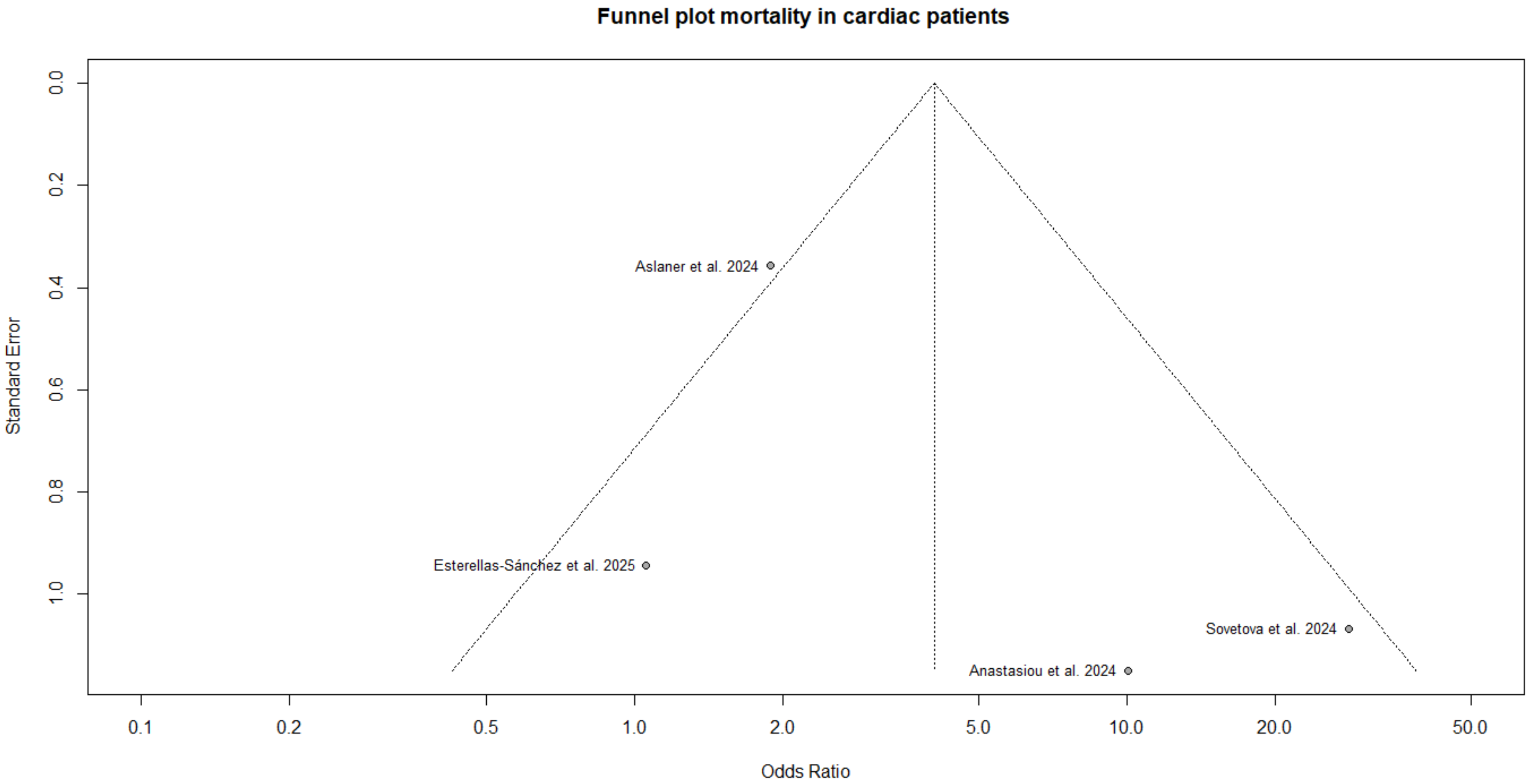
Funnel plot mortality in cardiac patients
Standard Error
0.0
0.2
0.4
0.6
0.8
1.0
Aslaner et al. 2024
Esterellas-Sánchez et al. 2025
Sovetova et al. 2024
Anastasiou et al. 2024
0.1
0.2
0.5
1.0
2.0
5.0
10.0
20.0
50.0
Odds Ratio

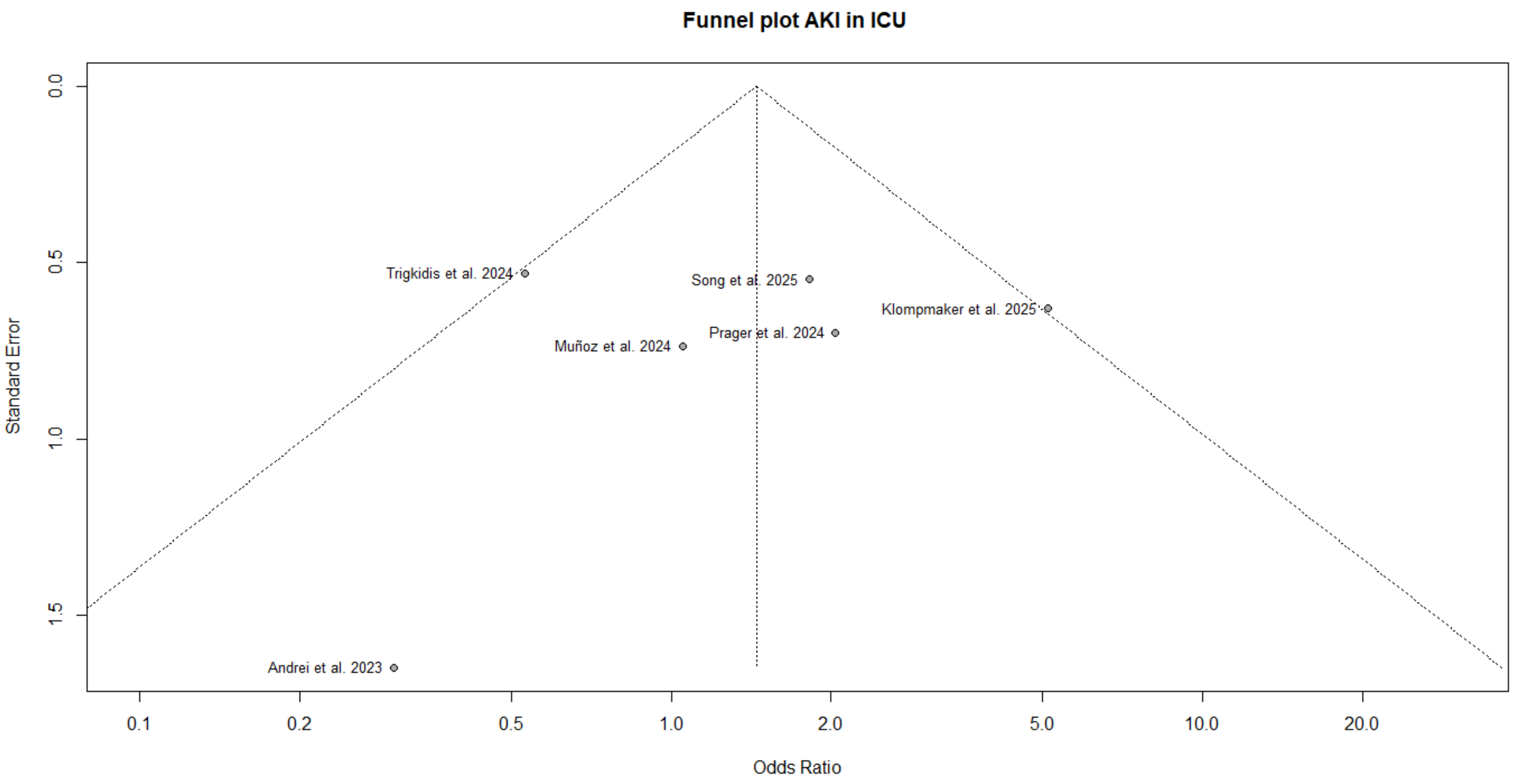
Funnel plot AKI in ICU
Standard Error
0.0
0.5
1.0
1.5
Trigkidis et al. 2024
Song et al. 2025
Klompmaker et al. 2025
Prager et al. 2024
Muñoz et al. 2024
Andrei et al. 2023
0.1
0.2
0.5
1.0
2.0
5.0
10.0
20.0
Odds Ratio

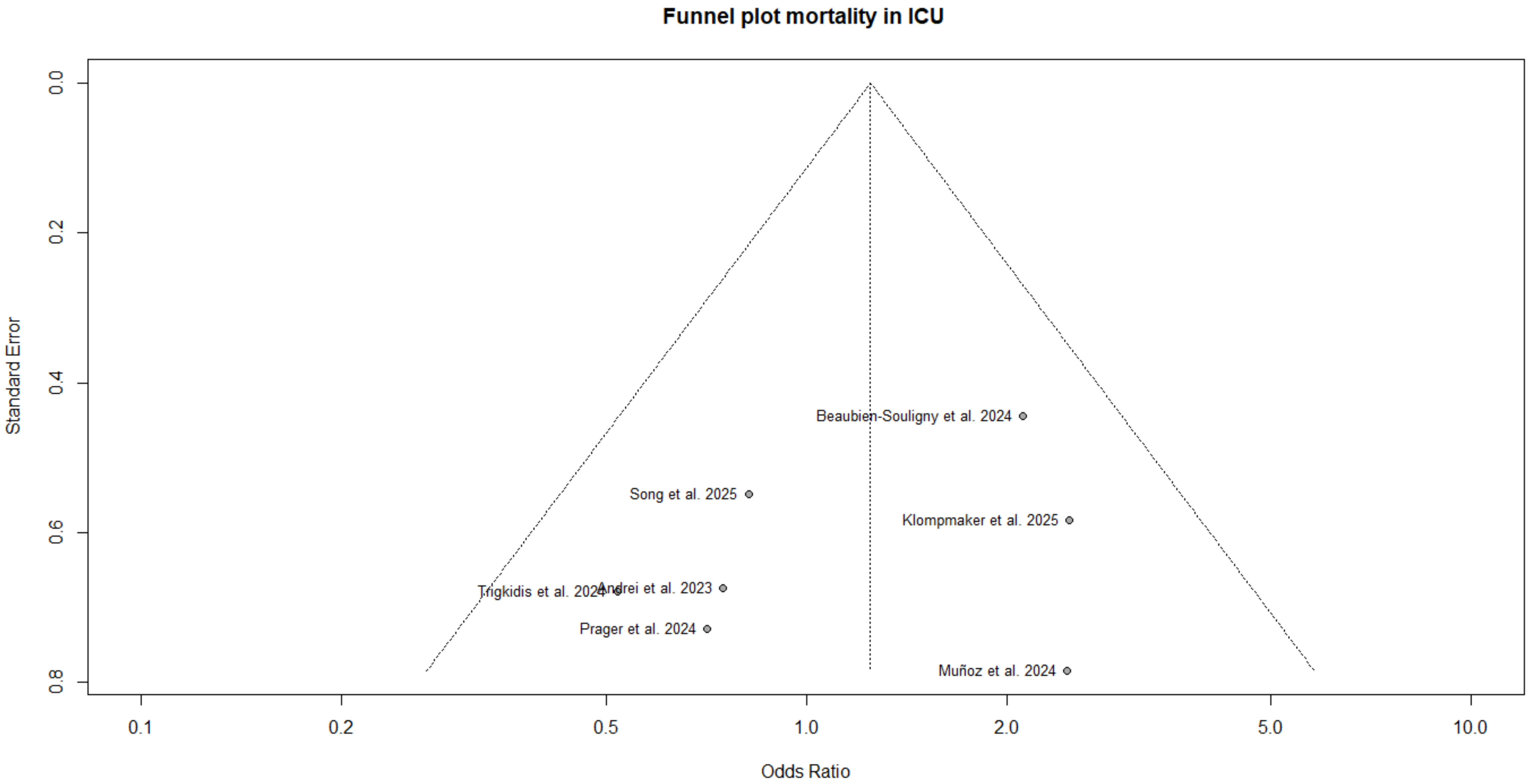

Supplementary table 4 Grade assessment cardiac AKI

Grade domain	Judgement	Concerns about certainty domains

Risk of bias	All studies included in this part of the meta-analysis had an overall low risk of bias.	No serious concerns
Inconsistency	Although one study had a confidence interval overlapping 1, the overall effect is in the same direction and consistent across studies	No serious concerns
Indirectness	All studies matched well with our research question, and investigated the association between VExUS and AKI	No serious concerns
Imprecision	There are 2 studies with a large confidence interval and one shows a not statistically significant effect. This might be due to the smaller population in these 2 studies. Moreover the amount of studies was somewhat limited. These concerns were minor but a decisions to downgrade was made to prevent overestimation of the certainty of the evidence.	Some concerns
Publication bias	No signs of publication bias were found using funnel plots.	No serious concerns

Supplementary table 5 Grade assessment cardiac mortality

Grade domain	Judgement	Concerns about certainty domains
Risk of bias	All studies included in this part of the meta-analysis had an overall low risk of bias.	No serious concerns
Inconsistency	The per patient analysis showed some fragility of the results and of one study driving the results.	Serious concerns
Indirectness	Although not all studies included had mortality as their main outcome it unlikely this had influenced their results.	No serious concerns
Imprecision	Low number of events and small study effect was noticed. There were several studies with such a low number of events that unreasonably wide confidence intervals were found	Serious concerns

Publication bias	No signs of publication bias were found using funnel plots.	No serious concerns

Supplementary table 6 Grade assessment ICU aki

Grade domain	Judgement	Concerns about certainty domains
Risk of bias	One of the included studies had a somewhat higher risk of bias, the other included studies had a low risk of bias and it was deemed unlikely to influenced the results of the analysis. Leave one out analysis confirmed this	No serious concerns
Inconsistency	Although point estimates varied in direction, heterogeneity was moderate (I^2 = 45%) and largely explained by wide confidence intervals and few events. We therefore did not downgrade for inconsistency.	No serious concerns
Indirectness	All included studies matched well with our research question	No serious concerns
Imprecision	We downgraded for imprecision due to few events and wide confidence intervals crossing the line of no effect and including both clinically important benefit and harm.	Serious concerns
Publication bias	No signs of publication bias were found using funnel plots.	No serious concerns

Supplementary table 7 Grade assessment ICU mortality

Grade domain	Judgement	Concerns about certainty domains
Risk of bias	One of the included studies had a somewhat higher risk of bias, the other included studies had a low risk of bias and it was deemed unlikely to influenced the results of the analysis. Leave one out analysis confirmed this.	No serious concerns

Inconsistency	Although point estimates varied in direction all confidence intervals overlap, this is largely explained by wide confidence intervals and few events. We therefore did not downgrade for inconsistency.	No concerns
Indirectness	The included studies matched well with our research question although mortality was not always the main outcome	No serious concerns
Imprecision	All included studies had wide confidence intervals most likely due to a smaller effect and lower event rates, which led to underpowered studies.	Serious concerns
Publication bias	No signs of publication bias were found using funnel plots.	No serious concerns

Supplementary table 8 Operator experience and used equipment of all included studies

Study	Operator experience	Used equipment
**Alday-Ramírez et al. 2025**	Trained cardiologist	GE Venue ultrasound (GE Healthcare, Illinois) phased array (1-5MHz
**Bhardwaj et al. 2023**	Trained intesivist >5 years experience	a Sonosite M Turbo machine, cardiac probe(1-5MHz)
**Klangthamneam et al. 2024**	Internal medicine resident and attending	Philips Lumify phased-array probe and the Kosmos TorsoProbe from Echo Nous.
**Menéndez-Suso et al. 2022**	Trained and certified pediatric intensivist	dge II; FUJIFILM Sonosite, Inc.; Bothell, WA, USA), with a 2-5 MHz (Sonosite C60) convex array transducer.

**Ruste et al. 2025**	unkown	Intellivue MX750 monitor (Philips Healthcare, Andover, MA, USA).
**Torres-Arrese et al. 2022**	ultrasound fellowship-trained internal medicine physician	A Mindray M7 diagnostic ultrasound system with Phased Array, Curvilinear and linear transducer (Mindray España, Madrid, Spain) and a Kosmos ultrasound handheld device (EchoNous, Redmond,WA, USA) were used in the study.
**Anastasiou et al. 2024**	All measurements were performed by 2 investigators with experience in hepatic and renal ultrasonography. Both of these investigators specialized in adult transthoracic echocardiography and underwent a 1-month part-time training at the radiology department of the hospital focused on renal and portal veins Doppler assessment.	phased-array transducers (Vivid E95 or Vivid S70, GE Healthcare).
**Andrei et al. 2023**	performed by a board certified physician	Philips Envisor ultrasound system (Affinity ultrasound system Philips Medical System, Suresnes, France)
**Aslaner et al. 2024**	these images underwent external review by one of the developers of the VExUS score.	Logiq S7 (GE Medical Systems, Milwaukee, Wisconsin) equipped with a 2–5 MHz convex transducer
**Beaubien-Souligny et al. 2024**	Experienced operators	an ultrasound machine with a phased array transducer and/or a convex array transducer
**Beaubien-Souligny et al. 2020**	Experienced operators	an ultrasound machine with a phased array transducer and/or a convex array transducer

**Bhardwaj et al. 2020**	intensivist who is experienced in ultrasound examination for at least 3 years.	Sonosite M Turbo machine using a curvilinear abdominal probe of 8–3 MHz
**Bhardwaj et al. 2025**	at least 3 years of experience	Philips CX50 model ultrasound machine.
**Esterellas-Sánchez et al. 2025**	two trained physicians performed image acquisition	Lumify R (Philips Iberica, Madrid, Espana) and Omega R (Esaote Espana S.A.U., Barcelona, Spain) devices, equipped with both phased-array and convex probes.
**Hassan et al. 2025**	Experienced physician	Unknown
**He et al. 2024**	Experienced radiologists	2011~3231663, Mindray M9, Shenzhen, China) equipped with a 5 MHz convex probe
**Khan et al. 2025**	Unknown	Unknown

**Klompmaker et al. 2025**	Mixed experience, reviewed by experienced operators	Philips CX 50 with phased array probe
**Landi et al. 2024**	Trained interal medicine fellows	Handheld (Kosmos EchoNous, Redmond, WA, USA)
**Li et al.2024**	Experienced operators (2 and 5 years)	Unknown
**Longino et al. 2024**	internal and emergency medicine residents with institutional training in ultrasound.	Unknown
**Lozano-Jiménez et al. 2025**	unknown	unknown
**Magin et al. 2024**	Medical students with POCUS training	Kosmos Ultraportable Torso-One device (Echonous Inc. Bothell WA) or a Fujifilm Sonosite LX device (Fujifilm Sonosite Inc. Bothell WA).
**Munoz et al. 2024**	Trained operators	Mindray M9 (Bio-Medical Electronics Co., Shenzhen, China) and SonoSite Edge II (Fujifilm Sonosite Inc., Bothell, WA, USA

**Natraj et al. 2024**	Unknown	Unkown
**Prager et al. 2024**	Trained operators	Mindray M9 machine (Mindray Bio-Medical Electronics Co., Ltd., Shenzen, China)
**Rodríguez-Moguel et al. 2024**	by resident pulmonology physicians who had at least 2 years of experience in ultrasound examinations	GE Venue ultrasound using a curvilinear abdominal probe of 1–5 MHz and a sectorial probe of 3 MHz
**Song et al. 2025**	Trained operators	CX-50 (Philips Healthcare, Amsterdam, The Netherlands),Venue Go™ (GE Healthcare, Wuxi City, China) and M9 (Mindray, Shenzhen, China) ultrasound devices
**Sovetova et al. 2024**	Unknown	Unknown
**Trigkidis et al. 2024**	Experienced operator	Vivid 7 (General Electric Healthcare, UK) ultrasound
**Utrilla-Alvarez et al. 2023**	Unknown	phased array sector probe at 2–3 MHz,(brand unknown)
**Viana-Rojas et al. 2023**	Trained cardiologist with experience	unknown

reviewer questions- Rmarkdown of model analysis

2025-12-28

```
library(lme4)

## Loading required package: Matrix

library(dplyr)

##
## Attaching package: 'dplyr'

## The following objects are masked from 'package:stats':
##
##     filter, lag

## The following objects are masked from 'package:base':
##
##     intersect, setdiff, setequal, union

library(purrr)
library(tibble)

glmer_diagnostics <- function(model, data, study_var, outcome_label = "") {

  cat("\n=============================\n")
  cat("Diagnostics for:", outcome_label, "\n")
  cat("=============================\n")

  ## --- Convergence ---
  cat("\nConvergence info:\n")
  print(model@optinfo$conv)

  ## --- Gradient ---
  grad_max <- max(abs(model@optinfo$derivs$gradient))
```

```r
cat("\nMax absolute gradient:", grad_max, "\n")

## --- Hessian ---
hess_eigen <- eigen(model@optinfo$derivs$Hessian)$values
cat("\nHessian eigenvalues:\n")
print(hess_eigen)

## --- Alternative optimizer ---
model_alt <- update(
  model,
  control = glmerControl(optimizer = "bobyqa")
)

cat("\nFixed effects (default optimizer):\n")
print(fixef(model))

cat("\nFixed effects (bobyqa optimizer):\n")
print(fixef(model_alt))

## --- Random effects distribution ---
ranef_vals <- ranef(model)[[study_var]][,1]

qqnorm(ranef_vals, main = paste("QQ plot of random intercepts:", outcome_label))
qqline(ranef_vals)

## --- ICC ---
var_study <- as.numeric(VarCorr(model)[[study_var]])
ICC <- var_study / (var_study + (pi^2 / 3))

cat("\nIntraclass Correlation Coefficient (ICC):", ICC, "\n")

## --- Leave-one-study-out analysis ---
studies <- unique(data[[study_var]])

loo_est <- map_dbl(studies, function(s) {
```

```r
    m <- update(model, data = data %>% filter(.data[[study_var]] != s))
    fixef(m)[2]   # assumes VEXUS grade 1 is coefficient 2
  })

  cat("\nLeave-one-study-out estimate range (log-odds):\n")
  print(range(loo_est))

  ## --- Random-effect variance stability ---
  loo_var <- map_dbl(studies, function(s) {
    m <- update(model, data = data %>% filter(.data[[study_var]] != s))
    as.numeric(VarCorr(m)[[study_var]])
  })

  cat("\nLeave-one-study-out random-effect variance range:\n")
  print(range(loo_var))

  invisible(list(
    gradient = grad_max,
    hessian_eigen = hess_eigen,
    ICC = ICC,
    loo_est_range = range(loo_est),
    loo_var_range = range(loo_var)
  ))
}

pooled_icu <- readRDS("pooled_icu.rds")
pooled_card <- readRDS("pooled_card.rds")

# random effects for per patient analysis ICU aki
perpatient.AKI.ICU <- glmer(aki ~ as.factor(vexus.grade) + (1 | studynumber), data = pooled_icu, family = binomial)


# random effects for per patient analysis ICU mortality
perpatient.mortality.icu <- glmer(mortality ~ as.factor(vexus.grade) + (1 | studynumber), data = pooled_icu, family
= binomial)
```

```
## random effects for per patient analysis aki in cardiac patients
perpatient.AKI.card <- glmer(aki ~ as.factor(vexus.grade) + (1 | studynumber), data = pooled_card, family = binomia
l)


## random effects for per patient analysis mortality in cardiac patients
perpatient.mortality.card <- glmer(mortality ~ as.factor(vexus.grade) + (1 | studynumber), data = pooled_card, fami
ly = binomial)

diag_mortality_icu <- glmer_diagnostics(
  model = perpatient.AKI.ICU,
  data = pooled_icu,
  study_var = "studynumber",
  outcome_label = "ICU AKI"
)

##
## ==============================
## Diagnostics for: ICU AKI
## ==============================
##
## Convergence info:
## $opt
## [1] 0
##
## $lme4
## list()
##
##
## Max absolute gradient: 0.0001250919
##
## Hessian eigenvalues:
```

```
## [1] 67.158971 32.719408 25.122441 16.018585  8.395481
##
## Fixed effects (default optimizer):
##             (Intercept) as.factor(vexus.grade)1 as.factor(vexus.grade)2
##              -0.1766045              -0.2484603              -0.1142356
## as.factor(vexus.grade)3
##               0.7749194
##
## Fixed effects (bobyqa optimizer):
##             (Intercept) as.factor(vexus.grade)1 as.factor(vexus.grade)2
##              -0.1766125              -0.2484583              -0.1142305
## as.factor(vexus.grade)3
##               0.7749191
```

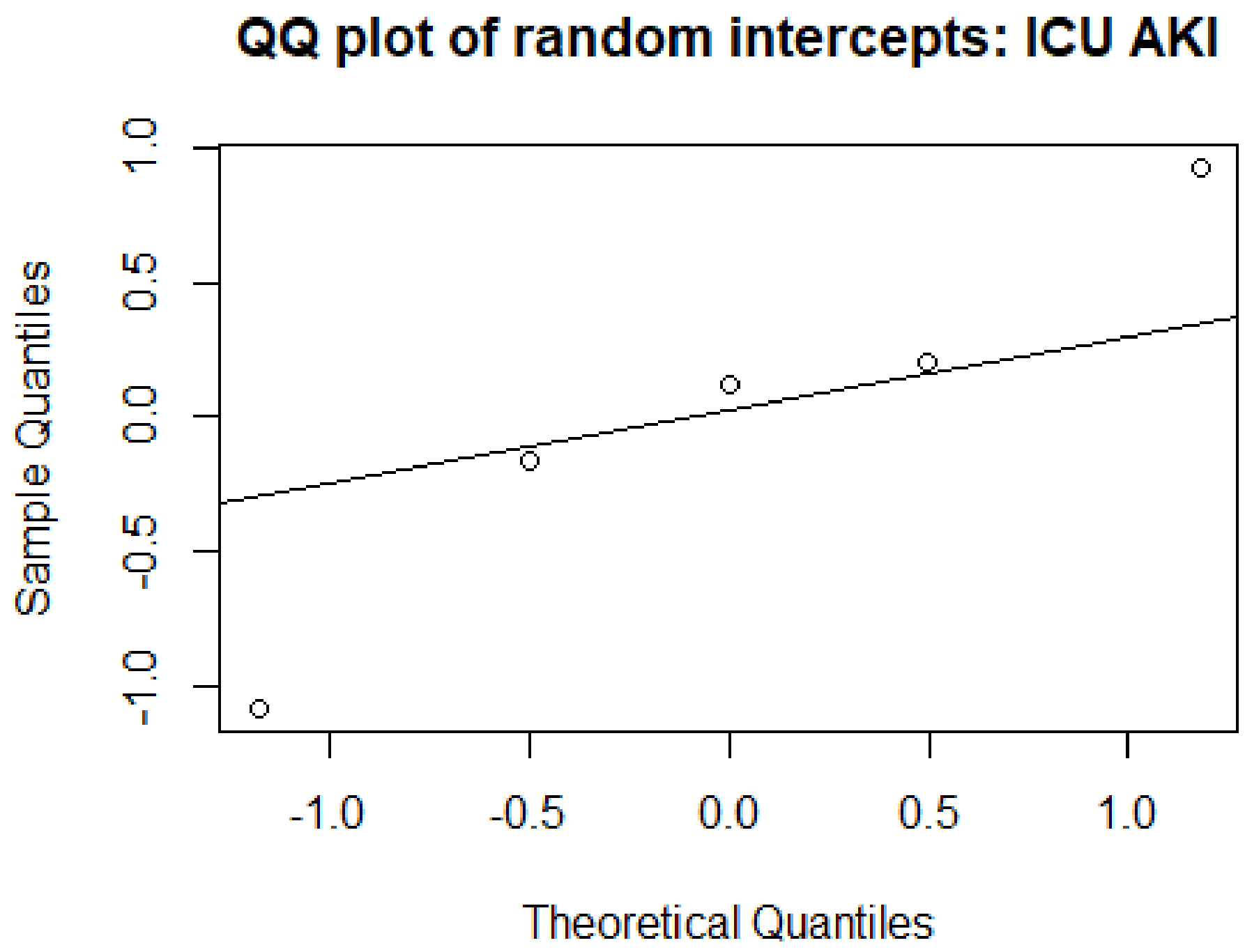


```
##
## Intraclass Correlation Coefficient (ICC): 0.1256549
##
## Leave-one-study-out estimate range (log-odds):
## [1] -0.40105418 -0.05001038
##
## Leave-one-study-out random-effect variance range:
## [1] 0.1506593 0.6325627
```

```
diag_mortality_icu <- glmer_diagnostics(
  model = perpatient.mortality.icu,
  data = pooled_icu,
```

```
  study_var = "studynumber",
  outcome_label = "ICU mortality"
)

##
## ==============================
## Diagnostics for: ICU mortality
## ==============================
##
## Convergence info:
## $opt
## [1] 0
##
## $lme4
## list()
##
##
## Max absolute gradient: 0.0003192491
##
## Hessian eigenvalues:
## [1] 150.214037 113.714268  48.044623  21.962627   9.715111
##
## Fixed effects (default optimizer):
##            (Intercept) as.factor(vexus.grade)1 as.factor(vexus.grade)2
##             -1.4205159               0.1319832               0.2015411
## as.factor(vexus.grade)3
##              0.8821341
##
## Fixed effects (bobyqa optimizer):
##            (Intercept) as.factor(vexus.grade)1 as.factor(vexus.grade)2
##             -1.4205268               0.1319954               0.2015611
## as.factor(vexus.grade)3
##              0.8821353
```

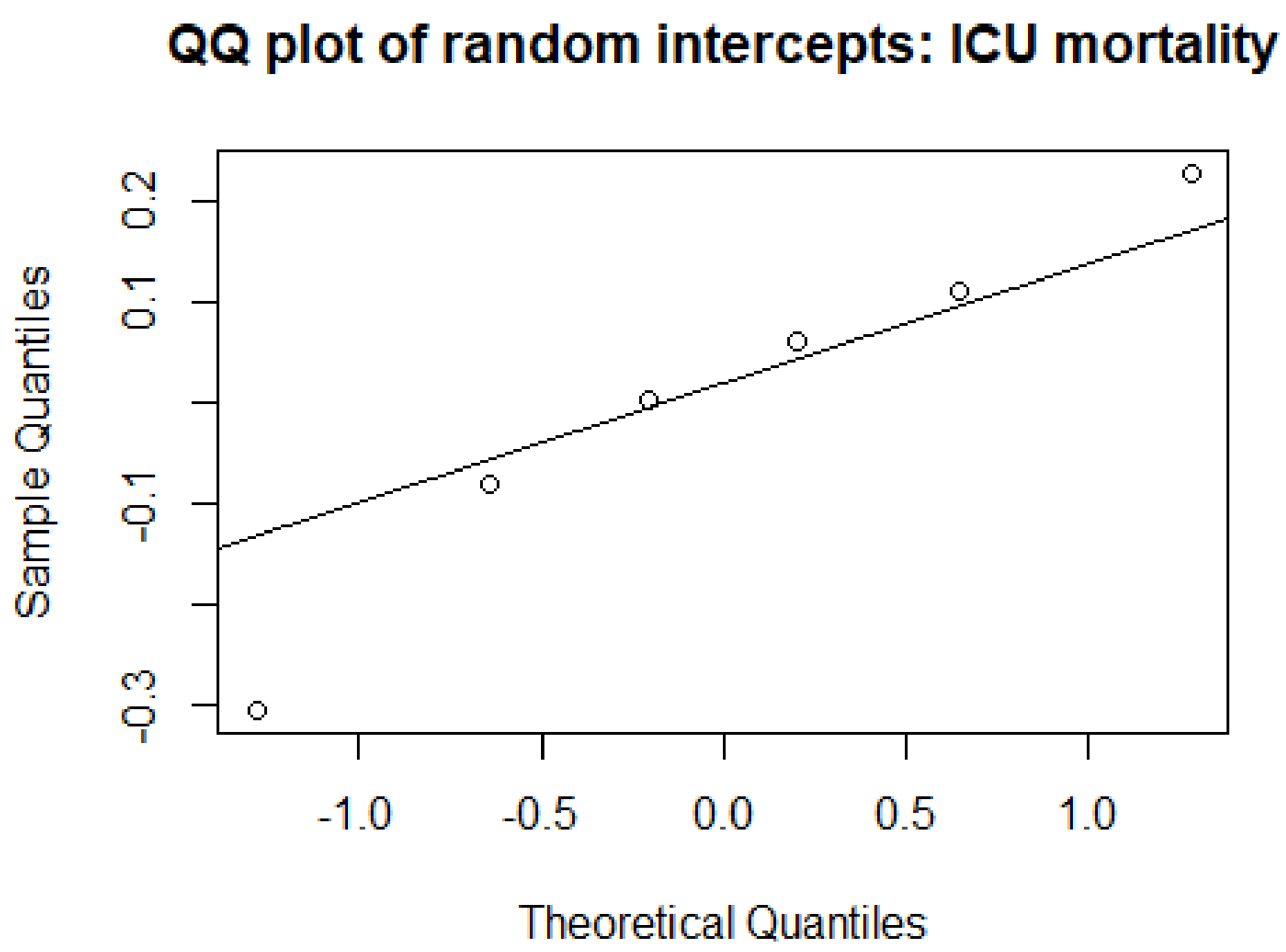


```
##
## Intraclass Correlation Coefficient (ICC): 0.01685834

## boundary (singular) fit: see help('isSingular')

##
## Leave-one-study-out estimate range (log-odds):
## [1] -0.2446830  0.3391826

## boundary (singular) fit: see help('isSingular')
```

```
##
## Leave-one-study-out random-effect variance range:
## [1] 0.000000 0.116982
```

```
diag_AKI_card <- glmer_diagnostics(
  model = perpatient.AKI.card,
  data = pooled_card,
  study_var = "studynumber",
  outcome_label = "AKI – cardiac patients"
)
```

```
##
## =============================
## Diagnostics for: AKI – cardiac patients
## =============================
##
## Convergence info:
## $opt
## [1] 0
##
## $lme4
## list()
##
##
## Max absolute gradient: 0.0004162644
##
## Hessian eigenvalues:
## [1] 185.45165 106.64955  42.93431  26.43245  13.31309
##
## Fixed effects (default optimizer):
##             (Intercept) as.factor(vexus.grade)1 as.factor(vexus.grade)2
##              -1.2620681               0.1261671               0.8550240
## as.factor(vexus.grade)3
##               2.0197987
##
```

```
## Fixed effects (bobyqa optimizer):
##               (Intercept) as.factor(vexus.grade)1 as.factor(vexus.grade)2
##                -1.2620692               0.1261614               0.8550367
## as.factor(vexus.grade)3
##                 2.0198019
```

QQ plot of random intercepts: AKI – cardiac patien

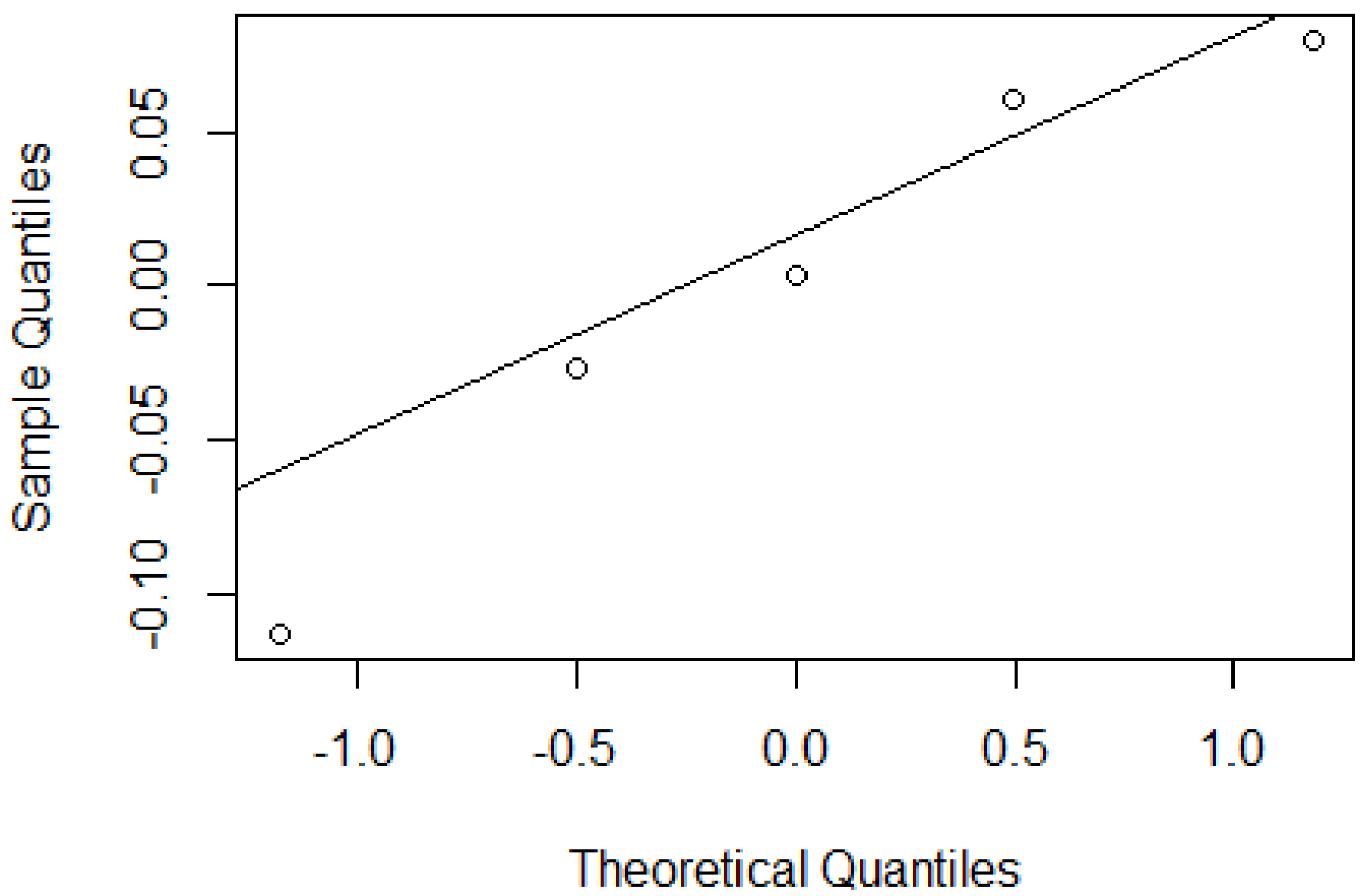


```
##
## Intraclass Correlation Coefficient (ICC): 0.005268926

## boundary (singular) fit: see help('isSingular')
```

```
##
## Leave-one-study-out estimate range (log-odds):
## [1] -0.01558271  0.34441952

## boundary (singular) fit: see help('isSingular')

##
## Leave-one-study-out random-effect variance range:
## [1] 4.153086e-15 3.476122e-02

diag_mortality_card <- glmer_diagnostics(
  model = perpatient.mortality.card,
  data = pooled_card,
  study_var = "studynumber",
  outcome_label = "Mortality – cardiac patients"
)

##
## ==============================
## Diagnostics for: Mortality – cardiac patients
## ==============================
##
## Convergence info:
## $opt
## [1] 0
##
## $lme4
## list()
##
##
## Max absolute gradient: 0.0002937759
##
## Hessian eigenvalues:
## [1] 36.072541 24.144805 14.189122 12.237923  5.488513
##
## Fixed effects (default optimizer):
```

```
##             (Intercept) as.factor(vexus.grade)1 as.factor(vexus.grade)2
##              -3.2227204               0.3279216               0.3267618
## as.factor(vexus.grade)3
##               1.8529063
##
## Fixed effects (bobyqa optimizer):
##             (Intercept) as.factor(vexus.grade)1 as.factor(vexus.grade)2
##              -3.2227244               0.3279237               0.3267736
## as.factor(vexus.grade)3
##               1.8529110
```

QQ plot of random intercepts: Mortality – cardiac pat

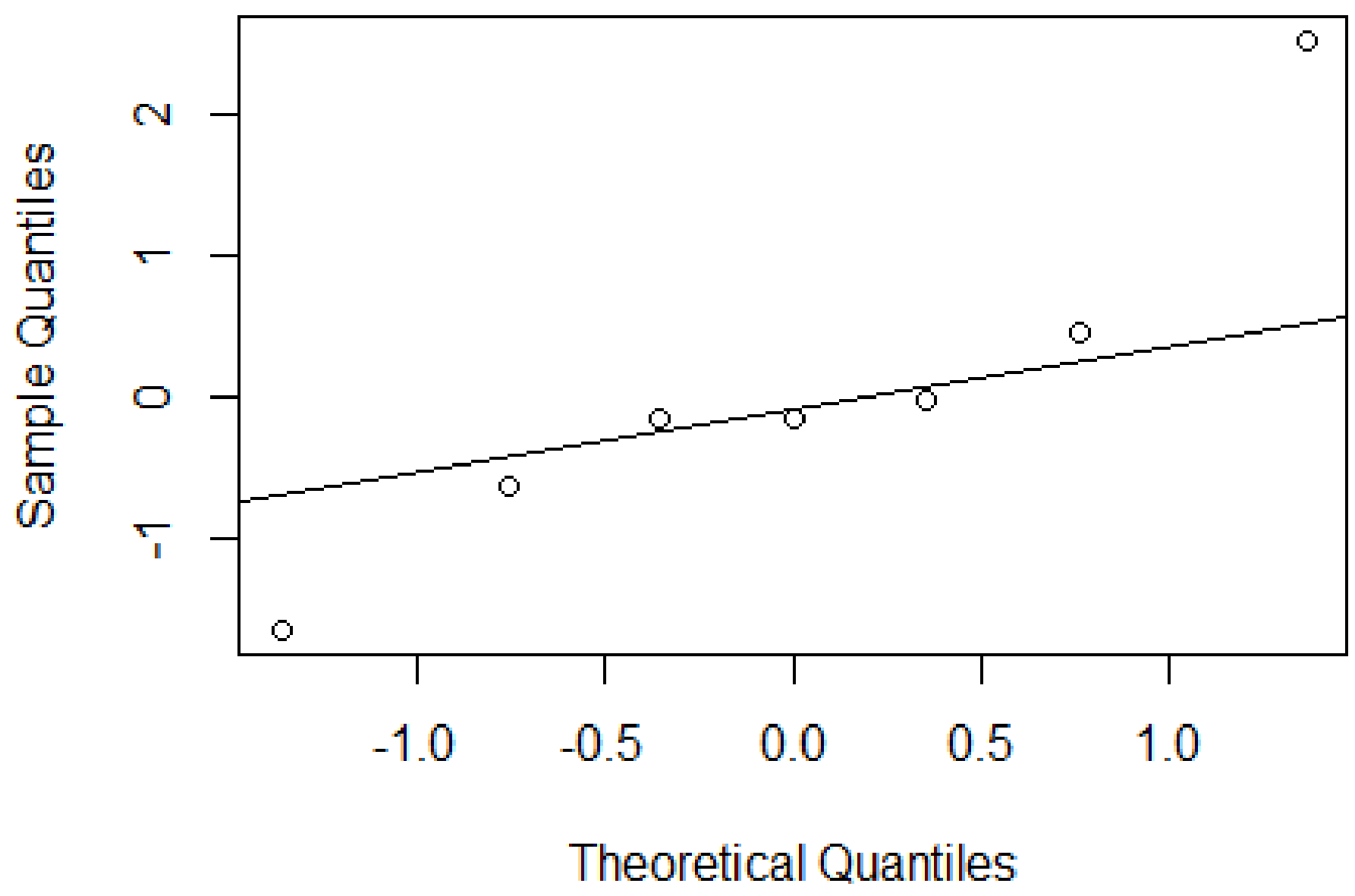

```
##
## Intraclass Correlation Coefficient (ICC): 0.3308551
##
## Leave-one-study-out estimate range (log-odds):
## [1]  0.2098363 17.3433973
##
## Leave-one-study-out random-effect variance range:
## [1] 0.3462629 1.9401316
```
